# Dynamic RSVP in Modern Networks for Advanced Resource Control with P4 Data Plane

**DOI:** 10.3390/s25072244

**Published:** 2025-04-02

**Authors:** Pin-An Pan, Wen-Long Chin, Yen-Chun Huang, Yu-Xiang Huang, Cheng-Hsien Yu

**Affiliations:** VLSI Signal Processing Laboratory, Department of Engineering Science, National Cheng Kung University, Tainan 701, Taiwan; xavier11012@gmail.com (P.-A.P.); u04tjp6123@gmail.com (Y.-C.H.); f131496@gmail.com (Y.-X.H.); aa0933533131@gmail.com (C.-H.Y.)

**Keywords:** software-defined networking (SDN), P4 language, resource reservation protocol (RSVP), Quality of Service (QoS), mininet, BMv2 software switch, wireless networks

## Abstract

This study focuses on leveraging the emerging Software-Defined Networking (SDN) technology, P4, to design a data plane for the Resource Reservation Protocol (RSVP) that can be applied in various scenarios, including both wired and wireless networks. This research explores the signaling mechanisms of the RSVP protocol, consolidates the data plane processing requirements, and ensures compliance with RSVP session Quality of Service (QoS) demands. Additionally, this study introduces the architecture, syntax, and external functionalities of the P4 language, which are utilized to develop the data plane required for RSVP-based resource reservation. Various parameters are pre-configured to enable the control plane to efficiently integrate RSVP reservation information into the data plane. Furthermore, Mininet is employed to create a virtual network topology, along with the BMv2 software switch, to evaluate whether the proposed system can fulfill RSVP’s end-to-end QoS guarantees. Different traffic transmission scenarios are examined to validate the system’s capability in accurately managing bandwidth allocation, latency, priority configuration, and packet counting for end-to-end QoS services.

## 1. Introduction

With the rapid development of the internet, the demand for Quality of Service (QoS) in network services has become a crucial issue. This is especially important in wireless network environments, where achieving low-latency, low-packet-loss, and high-bandwidth guarantees is essential for real-time communication applications such as voice calls and video conferencing. However, traditional IP networks operate on a best-effort basis, handling packets without finer-grained traffic differentiation or rate limiting. As a result, real-time communication applications often lack stable and guaranteed bandwidth.

Integrated Services (IntServ) aim to provide end-to-end QoS guarantees, with the Resource Reservation Protocol (RSVP) [1] serving as its core mechanism. RSVP enables end-to-end bandwidth and delayed reservation for specific traffic flows by coordinating packet processors along the entire data path. Due to its strict reservation mechanism, RSVP is more suitable for small-scale networks and is challenging to deploy in large-scale network environments. Despite its limited scalability and protocol compatibility, RSVP remains applicable in high-reliability, high-QoS dedicated networks.

P4 (Programming Protocol-Independent Packet Processors) [2] is an emerging Software-Defined Networking (SDN) technology that enables programmability in the network data plane. It allows for custom protocol definitions and complex packet forwarding logic tailored to different network scenarios, while also supporting the dynamic reconfiguration of forwarding rules. This flexibility makes P4 an ideal solution for future large-scale network applications, enabling efficient and adaptive network node deployment.

This study aims to explore the deployment of RSVP in an SDN environment and investigate how P4 can be leveraged to program its data plane, particularly in wireless network scenarios where dynamic resource allocation and QoS management are critical. This research focuses on RSVP packet parsing, traffic identification for resource reservation, bandwidth guarantees, delay management, priority configuration, and packet counting.

Given the unpredictable nature of wireless networks, where bandwidth fluctuations, interference, and varying latencies pose challenges, this study emphasizes how P4’s programmability can enhance RSVP’s adaptability to wireless environments. By dynamically adjusting bandwidth allocation, prioritization policies, and traffic differentiation, the proposed approach improves fine-grained traffic management and QoS assurance for wireless SDN-based networks. Through this work, our objective is to improve the feasibility of RSVP in SDN-driven wireless infrastructures, ensuring a more efficient use of network resources and stable end-to-end QoS provisioning in highly dynamic wireless communication environments.

This study makes the following key contributions:

- Integration of RSVP with SDN for Wireless Networks: We investigate how RSVP can be effectively deployed in an SDN environment, particularly in wireless network scenarios where dynamic resource allocation is essential;
- P4-Based Data Plane Implementation: We design and implement an RSVP-enabled data plane using P4, allowing fine-grained traffic management and real-time QoS provisioning;
- Dynamic Bandwidth and Delay Management: By leveraging P4’s programmability, we develop a mechanism to dynamically adjust bandwidth allocation and delay thresholds to adapt to varying wireless network conditions;
- Per-Flow RSVP Priority Configuration: We implement a mechanism that allows each RSVP flow to be assigned a different priority level, ensuring that high-priority flows receive preferential bandwidth and delay guarantees while lower-priority flows are still allocated fair network resources;
- Traffic Monitoring and Adaptive Control: A counter-based monitoring mechanism is implemented to provide real-time network status feedback to the control plane, enabling more accurate and adaptive resource reservations;
- Comprehensive Experimental Validation: We evaluate the effectiveness of our proposed system through real-world network topology simulations, demonstrating its feasibility and performance improvements in wireless SDN infrastructures.

This study is structured as follows. Section 2 introduces the P4 architecture and RSVP signaling mechanism. Section 3 reviews related work, discussing prior research and its relevance to our approach. Section 4 presents our proposed system architecture, explaining its key components and implementation. To verify the accuracy and functionality of the proposed design, Section 5 conducts multiple experiments. Notably, the experiments in this study utilize P4_16, the latest version of the P4 language, to ensure compatibility and extensibility. Finally, Section 6 summarizes our contributions and explores potential future research directions.

## 2. Background

### 2.1. Programming Protocol-Independent Packet Processors (P4)

P4 (Programming Protocol-Independent Packet Processors) was introduced in 2013 by a research team at Stanford University. It is a domain-specific programming language that enables software-defined control over network hardware processors, such as routers and switches. P4 allows developers to define packet forwarding rules and processing logic within the data plane while maintaining protocol independence.

P4 addresses the limitations of OpenFlow [2], another SDN technology that defines the communication protocol between SDN controllers and the data plane. Although OpenFlow provides packet parsing and lookup table matching, it supports only a limited set of network protocols and offers restricted actions for packet processing. As a result, any requirement for new network protocols requires a continuous expansion of OpenFlow’s capabilities. P4 overcomes these limitations by making the entire data plane programmable. It is designed with three key objectives:

(1) Reconfigurability—Developers can dynamically configure different data planes on network switches based on specific application requirements;
(2) Protocol independence—P4 allows users to define custom packet formats and header structures, eliminating dependencies on predefined network protocols;
(3) Target independence—P4 programs are hardware-agnostic, which means that they do not need to be rewritten for different hardware devices; instead, the compiler translates the P4 code into the appropriate hardware-specific instructions.

With these capabilities, future network architectures will become significantly more flexible, providing better traffic management, protocol adaptability, and extensibility. The programmability of P4 ensures that networks can efficiently accommodate evolving protocols and dynamic traffic demands.

#### 2.1.1. P4 Workflow

**Figure 1:**
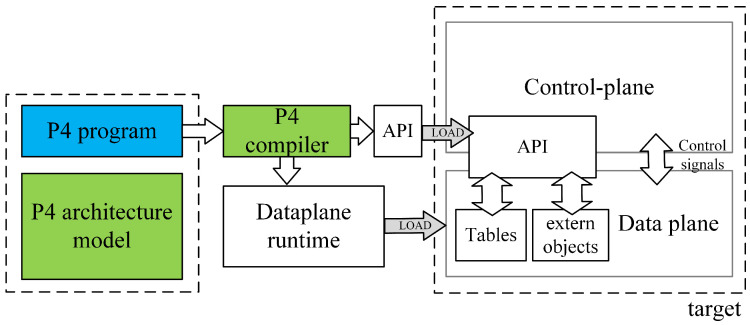
P4 workflow.

The working principle of P4 is illustrated in Figure 1. The workflow can be divided into two main components: the P4 program, provided by the user; and the P4 architecture model, the P4 compiler, and the target, provided by the manufacturer. A P4 programmer must design a P4 program based on the programmable blocks defined by the architecture provided by the manufacturer and utilize the architecture-specific function libraries supported by the target platform.

After the P4 program and P4 architecture files are processed by a specific compiler, two output files are generated: one defining the forwarding logic for the data plane configuration and another serving as an API for managing data exchange between the control plane and the data plane, such as parameters for flow table entries and extern functions. These files are then deployed onto the designated target device.

Following the principle of target independence, P4 programs can be implemented on various platforms, including programmable network interface cards (NICs), FPGAs, software switches, and hardware ASICs. By leveraging this workflow, P4 enables efficient and flexible deployment across different target platforms, making it a versatile solution for modern network architectures.

#### 2.1.2. P4 Architecture

The P4 architecture is provided by the manufacturer and must define programmable blocks such as Parser, Ingress, Egress, and Deparser. These definitions guide P4 programmers in designing P4 programs that meet the requirements of the target hardware. To ensure compatibility, the manufacturer must also provide a compiler that conforms to the architecture specifications.

Among the commonly used four programmable blocks, the Parser acts as a packet-header analyzer, extracting relevant fields from incoming packets and mapping them to specific protocol structures. The Ingress stage, responsible for match-action processing, associates packets with flow table entries, determines forwarding decisions, and implements packet forwarding logic, including traffic management functions. The Egress stage handles the final processing before packets exit the system, applying last-minute modifications to packet fields and enforcing QoS scheduling policies. The Deparser, which works in contrast to the Parser, reconstructs the final packet format by converting structured data back into its packet representation.

**Figure 2:**
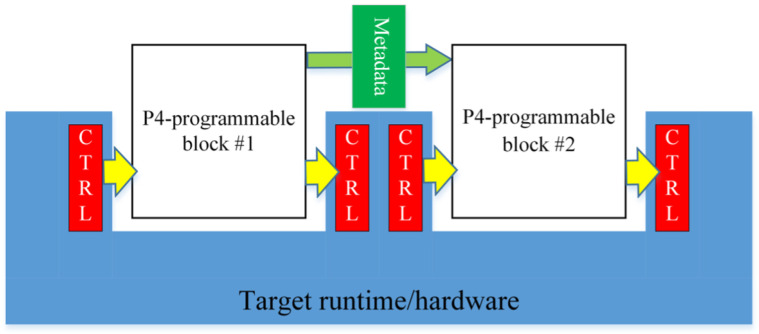
P4 architecture.

As illustrated in Figure 2, when two P4 programmable blocks are present, they can exchange user-defined metadata, which store necessary information during packet processing. Each programmable block communicates with the target hardware using input and output control signals, represented by intrinsic metadata. P4 programs can access intrinsic metadata from input control signals to retrieve hardware-defined information such as Ingress ports or timestamps. Additionally, they can modify intrinsic metadata to send control signals to the hardware, enabling operations such as setting the packet output port or marking packets for discard.

Beyond these components, the architecture-defined function libraries provide numerous external functions (externs), including meters, counters, and registers, which P4 programmers can call. Unlike the core P4 functions, externs are implemented by the underlying hardware platform, rather than within the P4 program itself. The function library defines externs using the extern keyword, specifying their syntax and required parameters. These externs offer P4 programs extended functionality beyond what can be achieved with native P4 programming.

Since different architectures define extern functions with unique parameters and return types, the declaration format and argument requirements may vary across architectures. Consequently, if a P4 program utilizes an extern function from one architecture, porting it to another architecture requires modifying the P4 program to match the new extern definitions.

### 2.2. P4 Meter

The metering mechanism in P4 follows the token bucket algorithm, implementing the Two-Rate Three-Color Marker (TRTCM) [3] standard. This algorithm uses several parameters:

- PIR (Peak Information Rate): The maximum allowable transmission rate when there is no network congestion;
- CIR (Committed Information Rate): The guaranteed minimum transmission rate during network congestion;
- PBS (Peak Burst Size): The maximum burst size allowed at the peak rate;
- CBS (Committed Burst Size): The guaranteed burst size allowed at the committed rate.

The metering process consists of two token buckets. The first bucket is replenished at a rate of PIR, and if the number of tokens exceeds the bucket size, the excess tokens are discarded. This bucket allows packets to be transmitted at the peak rate when the network is not congested. The second bucket is replenished at a rate of CIR, and, similarly, any excess tokens are discarded. This bucket ensures a minimum guaranteed rate even during congestion.

**Figure 3:**
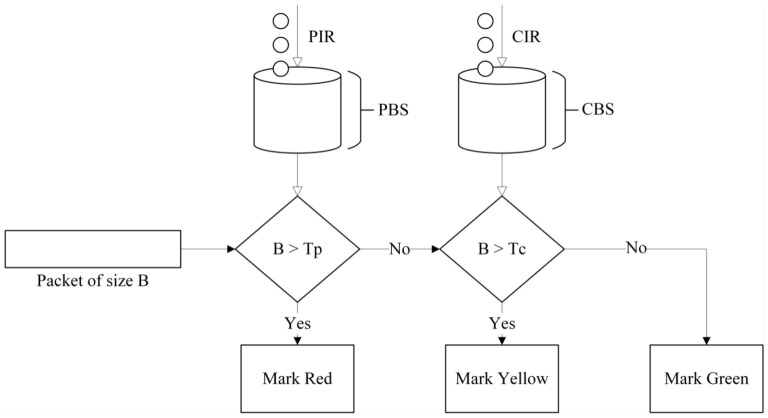
Two-Rate Three-Color Marker.

As illustrated in Figure 3, when a packet with size B arrives, it is processed based on token availability. If B can be deducted from the first bucket (Tp), the packet is considered within the PIR limit. If B is greater than Tp, the packet is marked as red. Otherwise, the system checks the second bucket (Tc). If B is greater than Tc, the packet is marked as yellow; if B is less than or equal to Tc, it is marked as green. In P4, this type of meter is referred to as byte mode, meaning that packets consume tokens based on packet size in bytes. If tokens are deducted at one per packet, the meter operates in packet mode. Finally, based on the three output classifications (red, yellow, green), the system determines whether the packet should be forwarded or dropped, effectively regulating network traffic.

### 2.3. Resource Reservation Protocol (RSVP)

The Resource Reservation Protocol (RSVP) was initially developed in the 1990s by the Internet Engineering Task Force (IETF) as part of the Integrated Services (IntServ) framework. Its specifications were defined in RFC 2205 [1], with the goal of supporting applications that require low latency and high bandwidth to meet strict Quality of Service (QoS) requirements.

IntServ is an end-to-end QoS mechanism that ensures precise resource allocation by reserving network resources at every packet-processing node along the data path. This strict reservation mechanism makes IntServ particularly suitable for high-reliability real-time applications, such as Voice over Internet Protocol (VoIP), video conferencing, online gaming, Virtual Reality (VR), and Data Center Interconnect (DCI). However, due to its resource-intensive nature, IntServ is only practical for small-scale networks and scenarios that demand stringent QoS guarantees.

In contrast, Differentiated Services (DiffServ), an alternative QoS framework, do not manage reservations for specific traffic flows. Instead, they apply traffic differentiation and congestion control at select network nodes only when congestion occurs. Due to the greater flexibility, DiffServ is more suitable for large-scale network deployments compared to IntServ.

RSVP was originally designed for Integrated Services Digital Network (ISDN) and Asynchronous Transfer Mode (ATM) network architectures. As network technologies and protocols evolved, RSVP has also been adopted across various network types, including IP networks, IP/MPLS networks, wireless networks, and Software-Defined Networking (SDN).

#### 2.3.1. RSVP Introduction

The RSVP protocol enables a host to reserve network resources along the path from the sender to the receiver to meet specific QoS requirements for an application. RSVP supports unidirectional packet reservation and must accommodate both unicast and multicast transmission scenarios.

**Figure 4:**
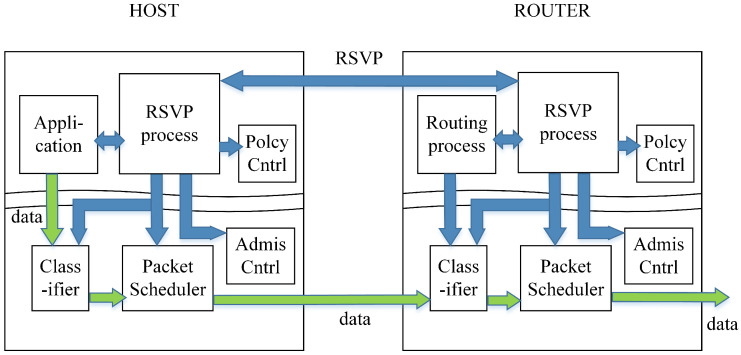
Mechanism of RSVP.

As illustrated in Figure 4, the RSVP signaling mechanism operates within the control plane of both the host and RSVP-enabled packet processors. The application requests the necessary QoS for data transmission, and this request is processed by the RSVP process, which forwards it to the Policy Control and Admission Control modules.

If the reservation request is approved, the RSVP process on the host and the packet processors pre-allocates the required network resources. The flow specification (flowspec) is then installed into the packet scheduler and packet classifier. During data transmission, packets passing through the classifier are checked to determine whether they belong to the RSVP-reserved flow. If they do, the packet scheduler processes them according to the reserved QoS parameters, ensuring proper prioritization and scheduling.

If the reservation request is rejected by Policy Control or Admission Control, the RSVP process returns an error message to the requesting receiver, indicating that the reservation could not be fulfilled.

#### 2.3.2. RSVP Workflow

To establish a resource reservation mechanism, RSVP transmits two types of messages: Path and Resv. If any network node along the transmission path does not support RSVP, these messages will be ignored, and the resource reservation process will not be completed.

**Figure 5:**
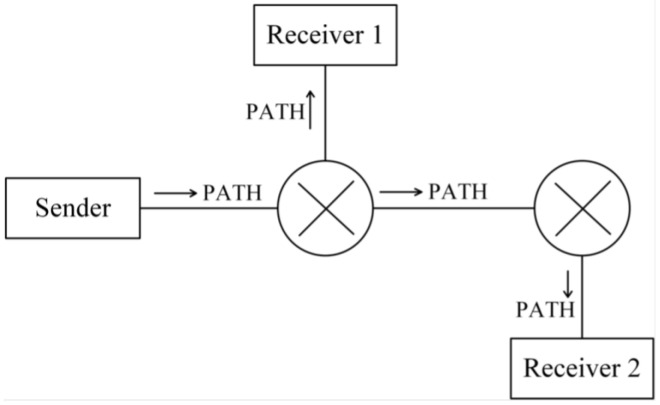
Transmission path of Path message.

To successfully reserve resources, the sender must first transmit a Path message, which follows the same unicast or multicast route as other transmitted data, as illustrated in Figure 5. The Path message contains three key components:

(1) Sender Template, which describes the type of message sent by the sender and is represented using a Filter Specification. It records information such as the IP address, TCP/UDP port, and protocol used by the sender for the reserved data, enabling the differentiation of traffic requiring reservation along the same link;
(2) Sender Tspec, which contains traffic characteristics of the sender’s data flow and allows network nodes to evaluate resource availability;
(3) Advertising Data (ADspec) refers to the One Path With Advertising (OPWA) information, which enables QoS-enabled network nodes along the route to advertise their supported services, available resources, and transmission characteristics. This advertisement helps the receiver’s application select an appropriate reservation specification.

**Figure 6:**
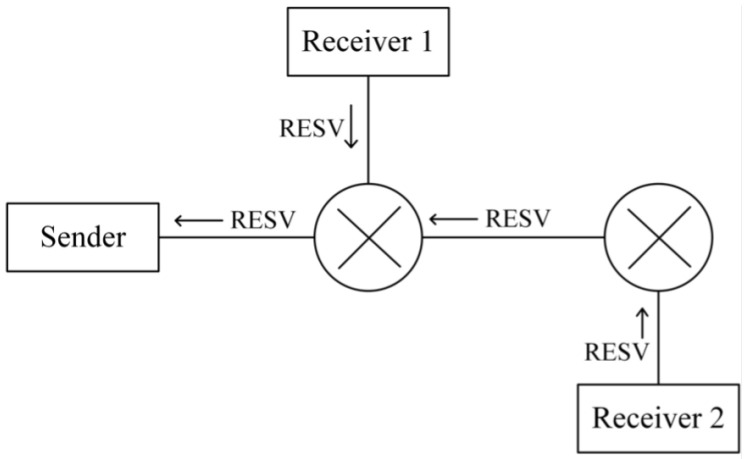
Transmission path of Resv message.

Once the Path message has been transmitted and network nodes along the route have processed the reservation-related information, the receiver initiates a Resv message to request resource reservation from each node, as illustrated in Figure 6.

Unlike the Path message, which is sent from the sender to the receiver, the Resv message travels in the opposite direction, from the receiver back to the sender. A key difference between these two messages is how their source and destination IP addresses are handled. The Path message has the sender’s IP address as its source and the receiver’s IP address as its destination. In contrast, the Resv message dynamically adjusts its source and destination addresses at each hop, following the reverse path of the Path message. This ensures that routers verify the correct routing of Resv messages and prevents packet loss caused by changes in network topology.

The Resv message contains a reservation specification (Rspec), which records the receiver’s QoS requirements and requests resource allocation at each network node. The network nodes then allocate resources according to the requested Rspec parameters, ensuring an end-to-end reservation that meets the receiver’s QoS demands.

In summary, RSVP enables precise resource reservation by using Path and Resv messages. The Path message, carrying Tspec, informs network nodes about the characteristics of transmission flow and resource consumption, while the Resv message propagates hop by hop, specifying the reservation requirements defined by the receiver’s Rspec. Through this signaling process, RSVP ensures precise and efficient resource allocation across the network.

## 3. Related Works

Ref. [2] introduces P4 (Programming Protocol-Independent Packet Processors) as a fully programmable language for packet processors. It was designed with three key principles: reconfigurability in the field, protocol independence, and target independence. P4 aims to address the limitations of OpenFlow [4], which functions only as a southbound interface between the control and data planes and supports only a predefined set of protocols. Although OpenFlow continues to expand its protocol support, it struggles to keep pace with the rapid evolution of network protocols. Ref. [5] leverages P4 programming for packet processors and employs P4Runtime as the control plane to dynamically define the forwarding rules. Furthermore, the study uses registers in the data plane to implement the automatic learning of forwarding tables for the Address Resolution Protocol (ARP). The authors compare the performance of standard metadata and non-standard metadata when using registers, which inspired this study to explore how RSVP forwarding logic can be implemented in the data plane using P4. Ref. [6] discusses the complexity of achieving QoS guarantees in traditional network switches. With the emergence of P4 and OpenFlow, researchers have found that P4 provides greater flexibility for implementing QoS services. In their study, the authors propose a P4-based bandwidth management switch, capable of classifying traffic based on its characteristics and applying bandwidth control and priority allocation. Furthermore, Ref. [7] points out that most commercial packet processors include meters and evaluates their efficiency. The study finds that, while meters effectively regulate UDP traffic, they face challenges when handling TCP traffic due to the Explicit Congestion Notification (ECN) mechanism, which conflicts with meter-based control. To address this issue, the authors propose a TCP-friendly meter, which they implement in the P4 data plane, achieving an increase in TCP flow rate compliance from 10% to 85%.

Several studies have explored different aspects of Software-Defined Networking (SDN), focusing on network performance measurement, QoS optimization, and implementation challenges [8,9,10]. Ref. [8] addresses the challenge of accurate link delay measurement, particularly in multi-hop environments, which is crucial for network management. Traditional methods struggle with precision, especially under congestion. To improve this, a P4-based approach is introduced, utilizing lightweight probe packets generated at the link edge and processed at the opposite end. The study demonstrates higher measurement accuracy and reliability compared to conventional tools like ping. Ref. [9] focuses on QoS management in SDN-based virtualized networks, highlighting the importance of dynamic load balancing across servers. When excessive requests are directed to a single server, this leads to higher end-to-end delay, reduced transfer rates, and bandwidth constraints. To address this, a Dynamic Active Sensing Server Load Management (DASLM) algorithm is proposed, enabling the SDN controller to efficiently distribute loads based on throughput, response time, and RTT, reducing delays and improving bandwidth utilization. Ref. [10] conducts a comprehensive review of SDN deployment challenges, categorizing research into seven key areas, including network verification, flow rule installation, security, memory management, SDN simulation tools, programming languages, and controller platforms. The study highlights the importance of network testing to prevent inefficiencies and ensures consistent flow rule installation during policy changes. It also underscores the role of SDN simulation tools in validating network applications before real-world deployment.

Numerous studies have focused on the QoS capabilities of P4 [11,12,13]. Ref. [11] focuses on network virtualization, allowing multiple telecom users to share the same physical network infrastructure without interfering with the virtual networks (VNs) of others. The authors introduce a Multicolor Marker (MCM) approach, along with two bandwidth-sharing models: Remaining Bandwidth Sharing (RBS) and Remaining Bandwidth Non-Sharing (RBNS). Ref. [12] presents P4-PSFP (Per-Stream Filtering and Policing), a traffic filtering and policing mechanism defined in Time-Sensitive Networking (TSN). This mechanism ensures controllable latency in time-sensitive applications while preventing packet loss due to network congestion. The study implements PSFP in P4 and deploys it on a hardware switch to evaluate its performance. Ref. [13] introduces P4-TINS (P4-driven Traffic Isolation for Network Slicing) and highlights the lack of flexibility in OpenFlow. The authors adopt P4 as the basis of their system, enabling the slicing of the network to isolate different types of traffic and manage the allocation of bandwidth for each type independently. They evaluated their design using the ONOS controller and commercial off-the-shelf (COTS) P4 switches.

This study is inspired by [14], which comes from the Networked Systems Group (NSG), focusing on network system design, implementation, and optimization. The authors use the P4-utils library to write control plane configuration scripts in Python 3.13.1, simulating the RSVP signaling mechanism in the control plane. Meanwhile, the data plane is configured using MPLS packet forwarding logic. In contrast, this research aims to design RSVP packet forwarding logic in the P4 data plane for IP networks and explore how P4 capabilities can meet RSVP’s QoS requirements for the data plane.

## 4. System Architecture

Based on the RSVP signaling mechanism described in Section 2, once the control plane completes the resource reservation communication, it determines how to identify RSVP traffic and the required QoS parameters. These configurations are then passed down to the data plane, allowing it to classify RSVP flows and enforce traffic management rules accordingly.

Since P4 is a fully programmable language for data plane implementation, it can be used to realize the required functionality of RSVP. Therefore, this study focuses on leveraging the practical SDN technology of P4 to implement the RSVP-enabled data plane. In order for a P4-based packet processor to support different network protocols, the protocol-specific logic must be explicitly defined in P4. This study categorizes the RSVP data plane functionalities into the following key components:

(1) Parsing RSVP packets;
(2) Using RSVP signaling information to identify RSVP sessions by extracting specific field values;
(3) Applying RSVP-based QoS guarantees to specific RSVP sessions according to the received signaling information;
(4) Monitoring network device interfaces to assess overall network traffic conditions.

The following sections describe how this study utilizes P4 programming to implement the required RSVP packet processing logic in the data plane. Additionally, the accessibility and capabilities of P4 tools for packet processing are discussed. The key implementation components include the following:

(1) Operation logic of the packet parser;
(2) Match-action logic for packet processing;
(3) End-to-end traffic management mechanisms;
(4) End-to-end delay guarantees;
(5) Network traffic monitoring and feedback to the control plane.

### 4.1. System Architecture

The architecture adopted in this study is V1Model, a fundamental and widely used P4 architecture supported by BMv2 (Behavioral Model v2). The choice of V1Model is primarily due to its versatility and broad applicability. BMv2 serves as a software-based network processor designed for P4 program development, experimentation, and debugging. It supports P4Runtime, enabling table entry management, interaction with standard metadata, and control–data plane communication simulation.

V1Model includes the v1model.p4 library, which defines a set of standard metadata used in this study. The key metadata fields include ingress_global_timestamp and egress_global_timestamp, which help to measure packet delay across network nodes, as well as priority and egress_port. Additionally, V1Model provides several extern functions, including meter, counter, and mark_to_drop, which are utilized in this study.

**Figure 7:**
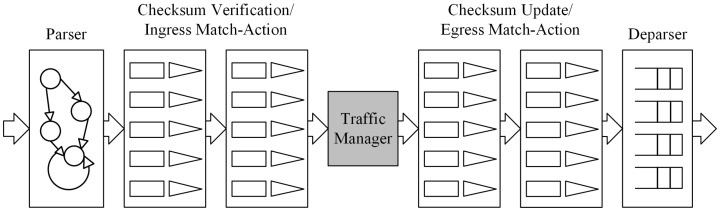
V1Model.

As illustrated in Figure 7, the V1Model processing pipeline consists of the following programmable blocks:

(1) Parser—A finite-state machine (FSM) responsible for packet-header parsing;
(2) Ingress Match-Action—Performs match-action table lookups and checksum validation for incoming packets;
(3) Egress Match-Action—Handles match-action lookups and checksum updates for outgoing packets;
(4) Deparser—Reconstructs the packet header and payload before transmission.

Additionally, V1Model contains a Traffic Manager, which is a non-programmable block. This means that P4 programmers cannot modify its internal functionality but can specify rules that dictate how it processes packets. From a P4 programming perspective, the Traffic Manager operates as a black box.

### 4.2. Parser Design in P4

In P4, the Parser operates as a finite-state machine (FSM), where the initial state is start and the final state is accept. The packet_in extern introduced in the Parser Block indicates that an external platform must provide an interface for receiving incoming packets. When a packet is received, it is assigned to a variable and processed using the .extract() function, which extracts the packet’s data into a predefined data structure declared by the programmer. The bits are sequentially extracted from the packet header, and transition select is used to evaluate specific field values and determine the next state transition.

**Figure 8:**
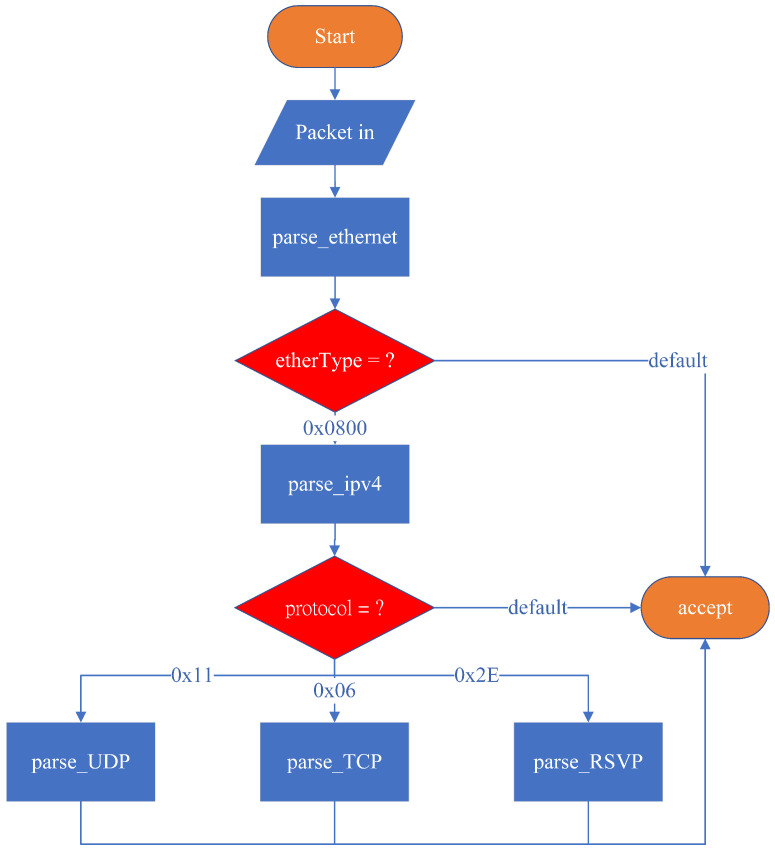
Parser workflow.

As illustrated in Figure 8, the system’s initial state is set to start by default. The parser then transitions to parse_ethernet, where it extracts the Ethernet header. Since this system is designed to support only IPv4, it recognizes packets where the EtherType field is 0x0800 (IPv4). If the EtherType value is 0x0800, the parser transitions to parse_ipv4; otherwise, it transitions to accept, meaning that no further header extraction is performed, and the remaining bits are treated as payload.

In the parse_ipv4 state, the protocol field is examined to determine the packet type. The system only supports three specific protocols for special processing: TCP, UDP, and RSVP. The state transitions are as follows:

(1) If the protocol field is 0x11 (UDP), the parser transitions to parse_UDP, extracting packet bits into the UDP header data structure;
(2) If the protocol field is 0x06 (TCP), the parser transitions to parse_TCP, extracting packet bits into the TCP header data structure;
(3) If the protocol field is 0x2E (RSVP), the parser transitions to parse_RSVP, extracting packet bits into the RSVP header data structure;
(4) If the protocol field does not match any of these values, the parser transitions to accept, where the packet is received, and the remaining bits are treated as payload.

This design outlines the complete packet parsing process for the system, allowing it to accurately parse RSVP packets and forward the extracted information to the control plane. After processing Path and Resv signaling messages, the P4 packet processor is configured to reserve resources accordingly. TCP and UDP packets, representing general traffic flows, are later analyzed to determine whether they require reservation-based control mechanisms for traffic management and resource allocation.

### 4.3. Ingress Match-Action Design in P4

**Figure 9:**
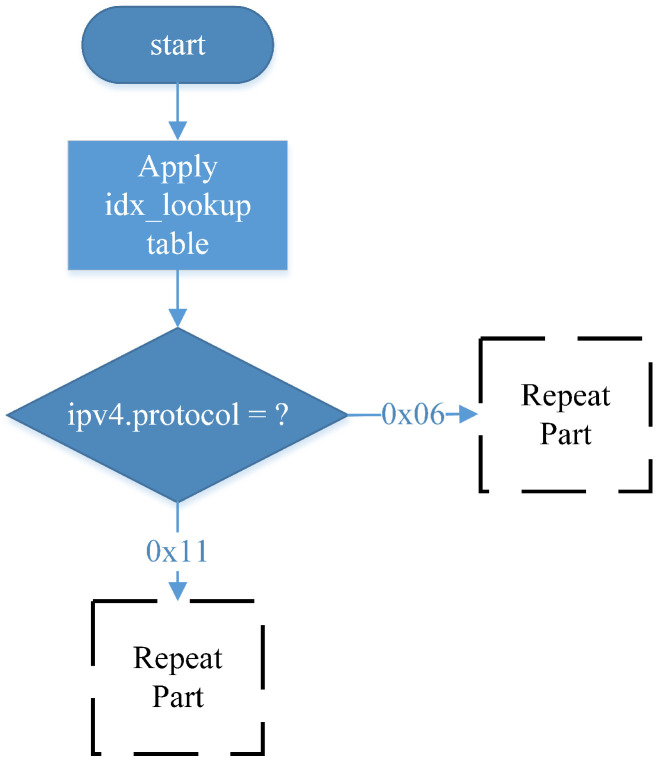
Ingress match-action workflow 1.

In the RSVP-compliant data plane design, as Figure 9, the system first queries the idx_lookup table, which matches packets based on their destination IP address. This allows the P4 packet processor to determine the appropriate output port for forwarding the packet. The retrieved port_id is then used as the identifier for a rate-limiting meter assigned to each Egress port. This meter does not limit RSVP-reserved traffic but instead allocates the remaining bandwidth to non-RSVP traffic, ensuring that unreserved traffic does not consume network resources designated for RSVP flows. The meter ID is derived from the output port ID, as each port has an independent link capacity. Additionally, port_id is used in a counter assigned to each port to monitor total resource utilization, which is then reported back to the control plane. The system then determines whether the incoming packet is TCP or UDP. Since these two protocols have different traffic characteristics, their traffic parameters are configured separately, and the lookup fields for identifying RSVP sessions differ. However, once a packet has been processed by the meter, the subsequent handling logic is identical, as illustrated in the repeat part of the diagram.

**Figure 10:**
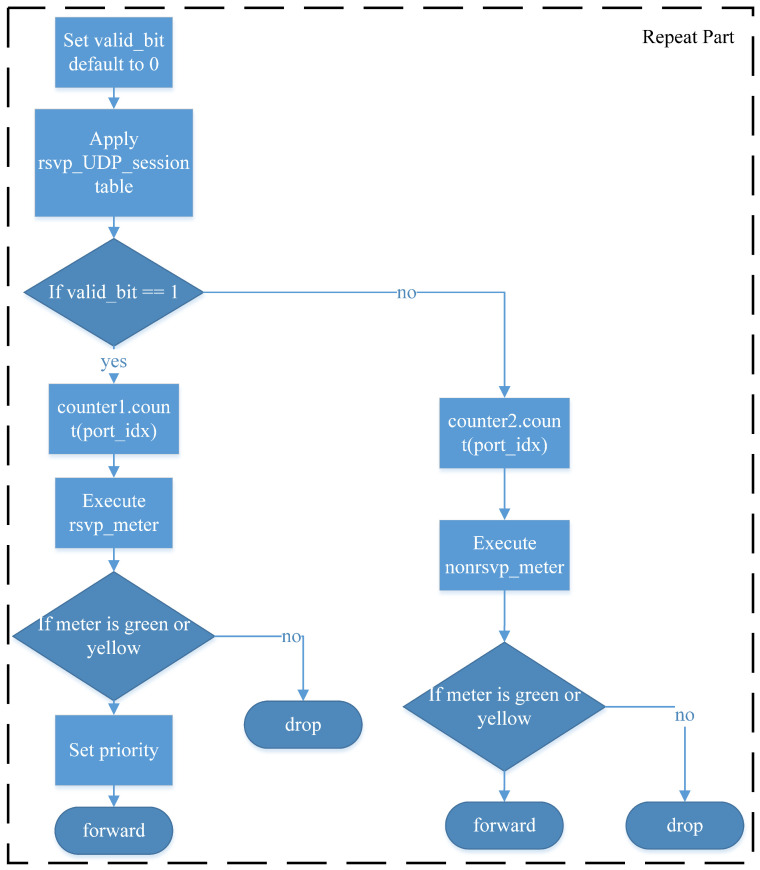
Ingress match-action workflow 2.

The subsequent workflow is illustrated in Figure 10. To differentiate RSVP-reserved flows, the system utilizes a valid_bit as a programming technique to store the RSVP reservation status for subsequent packet processing. Initially, valid_bit is set to invalid. When a UDP packet arrives, the rsvp_UDP_session table is queried using destination IP address, IP protocol, and UDP destination port, which are RSVP session identifiers. If a matching entry is found, the system retrieves the RSVP session ID, sets valid_bit to valid, and applies the configured priority settings. Similarly, for TCP packets, the rsvp_TCP_session table is queried using destination IP address, IP protocol, and TCP destination port. If a matching entry exists, the system extracts the RSVP session ID, sets valid_bit to valid, and retrieves the priority settings.

The system then checks the value of valid_bit. If valid_bit is valid, the system uses a counter to track both the number of packets and bytes to check whether the reserved resource allocation has been met. Moreover, the packet is processed using the rsvp_meter, where the RSVP session ID serves as the meter identifier. Since RSVP ensures end-to-end resource reservation, the same session ID is used across multiple network nodes, ensuring consistent reservation enforcement across all hops. The meter operates based on the Two-Rate Three-Color Marker (TRTCM) algorithm. The processing results are categorized as follows:

(1) Green: The packet is guaranteed to be forwarded;
(2) Yellow: The packet is forwarded on a best-effort basis, allowing for burst traffic when excess resources are available;
(3) Red: The packet exceeds the reserved resources and is discarded, as no additional bandwidth is available.

If a packet is classified as green or yellow, the priority settings defined by the control plane for that RSVP session are applied to the outgoing packet. Finally, the Ethernet source address, destination address, and IPv4 TTL are updated, and the packet is forwarded to the designated Egress port. If the packet is classified as red, it is dropped using standard_metadata settings.

When valid_bit is invalid, the system uses a counter to track both the number of packets and bytes for network resource monitoring, reporting the statistics to the control plane. This information allows RSVP to assess network usage and optimize reservation decisions. Finally, the system executes nonrsvp_meter, where the meter ID corresponds to each Egress port in the P4 packet processor. This meter ensures that non-RSVP traffic does not consume bandwidth reserved for RSVP sessions. The available bandwidth for non-reserved traffic is determined by subtracting the reserved bandwidth from the total link capacity. This means that the remaining capacity, which is not allocated to RSVP-reserved flows, can be utilized by general traffic. The metering results for non-RSVP traffic are handled in the same manner as RSVP traffic, following the green–yellow–red logic:

(1) Green packets are always forwarded;
(2) Yellow packets are forwarded opportunistically, depending on available bandwidth;
(3) Red packets are discarded.

Since non-RSVP traffic does not have a reserved priority, no priority adjustments are made. The system then updates the Ethernet source and destination addresses, decrements the IPv4 TTL, and forwards the packet to the designated Egress port. If the packet is classified as red, it is dropped using standard_metadata settings.

Table 1 summarizes all lookup tables applied within the Ingress block, which utilizes four lookup tables, each serving a distinct function:

(1) idx_lookup—This table determines the Egress port for an incoming packet by performing a lookup based on the destination IP address. The set_port_idx action assigns the retrieved result to a programmer-defined variable, which is subsequently used as the ID for nonrsvp_meter and the counter;
(2) rsvp_UDP_session—This table identifies whether an incoming UDP packet belongs to an RSVP-reserved flow. The lookup is based on three RSVP session parameters: destination IP address, IP protocol, and UDP destination port. If a matching entry is found, the table returns the vali_bit, priority, and session ID. The set_param action assigns these values to programmer-defined variables, which are then used to configure packet priority and the rsvp_meter ID;
(3) rsvp_TCP_session—This table identifies whether an incoming TCP packet belongs to an RSVP-reserved flow. The lookup fields are destination IP address, IP protocol, and TCP destination port, which define an RSVP session. If a matching entry exists, the table returns the valid_bit, priority, and session ID. As with rsvp_UDP_session, the set_param action assigns these values to programmer-defined variables, which are subsequently used for priority configuration and rsvp_meter ID assignment;
(4) routing_table—This table retrieves the necessary forwarding information for packet transmission. The lookup is performed using the destination IP address, returning the corresponding destination Ethernet address and Egress port. The ipv4_forward action updates the destination MAC address, sets the Egress port using standard_metadata, and assigns the source MAC address as the packet’s previous destination MAC address. Finally, the IPv4 TTL field is decremented by 1 to reflect the forwarding process.

**Table 1:** Tables applied in the Ingress match-action.

Tables	Keys	Actions
idx_lookup	Destination IP address	set_port_idx
rsvp_UDP_session	destination IP address, IP protocol, UDP destination port	set_parm
rsvp_TCP_session	destination IP address, IP protocol, TCP destination port	set_parm
routing_table	destination IP address	ipv4_forward

### 4.4. Egress Match-Action Design in P4

Transmission delay is typically calculated by dividing the total transmitted bytes by the link bandwidth, which determines the total time required for a packet to travel from one network node to another. However, since P4 programs are independently executed on each network processor, P4 cannot control or guarantee transmission delay across multiple nodes. Instead, P4 can be used to monitor and manage in-device processing delay, which is influenced by packet processing time and queuing time within the hardware device. Generally, in-device delay is much smaller compared to transmission delay.

**Figure 11:**
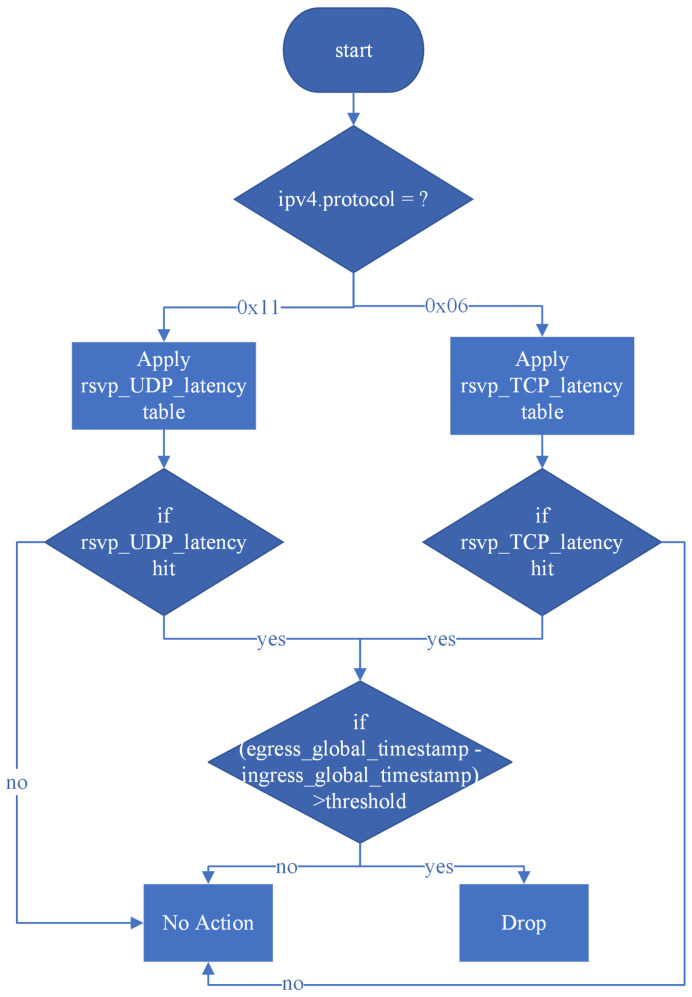
Egress match-action workflow.

To measure in-device delay, the system retrieves the Egress timestamp of the network processor and performs the calculation in the Egress match-action stage. As illustrated in Figure 11, the system first determines whether the L4 transport protocol is TCP or UDP, as this affects the lookup fields in the table. If the IPv4 protocol field indicates UDP with a value of 0x11, the system queries the rsvp_UDP_latency table. If the protocol field indicates TCP with a value of 0x06, the system queries the rsvp_TCP_latency table. After performing the table lookup, the system verifies whether a corresponding entry exists. If a matching entry is found, the packet is identified as an RSVP flow, and the corresponding latency threshold is retrieved. If no entry is found, the packet is determined to be non-RSVP traffic, and no further processing is applied.

The system then evaluates whether the in-device delay exceeds the RSVP-defined threshold. The delay is calculated by subtracting the Ingress timestamp from the Egress timestamp. If the calculated delay surpasses the predefined threshold, the packet is dropped. Otherwise, if the delay remains within the acceptable range, the packet proceeds without any modifications.

Table 2 summarizes all lookup tables applied in the Egress match-action stage of the system:

(1) rsvp_UDP_latency—Queries the latency threshold for UDP traffic using destination IP address, IP protocol, and UDP destination port as lookup fields. The set_latency_threshold action stores the retrieved latency threshold for use within the program;
(2) rsvp_TCP_latency—Queries the latency threshold for TCP traffic using destination IP address, IP protocol, and TCP destination port as lookup fields. The set_latency_threshold action stores the retrieved latency threshold for use within the program.

**Table 2:** Tables applied in the Egress match-action.

Tables	Keys	Actions
rsvp_UDP_latency	destination IP address, IP protocol, UDP destination port	set_latency_threshold
rsvp_TCP_latency	destination IP address, IP protocol, TCP destination port	set_latency_threshold

## 5. Experiments and Discussion

This chapter verifies whether the proposed system can achieve end-to-end QoS resource reservation in a multi-node network topology as expected. The chapter first introduces the experimental environment and computing devices used in the setup. It then details the experimental methodology and demonstrates the QoS resource reservation performance under different transmission scenarios. Finally, the results are analyzed using data and graphical representations to evaluate the system’s effectiveness.

### 5.1. Environment Setup

Here, we first introduce Mininet. Mininet is a network emulation tool that simulates switches and hosts using Linux Namespaces and virtual Ethernet links (veth), allowing all network nodes to run on a single physical machine without requiring multiple physical devices. In this emulation environment, the seven switches are instances of the BMv2 software switch, responsible for executing the P4 data plane, processing packet forwarding, and managing resources. The five hosts are independent network namespaces created by Mininet, simulating standalone devices capable of sending and receiving traffic for QoS testing.

These hosts do not run a complete, independent operating system but instead share the Linux kernel of the host machine. However, each host maintains its own network stack, process space, and filesystem view, making it behave like an independent machine capable of executing network commands and testing tools such as ping and iperf. This design enables realistic network behavior while minimizing resource overhead. By using Mininet, this study provides a flexible testing environment to evaluate the performance of RSVP-based resource reservation and bandwidth management in the P4 data plane without relying on physical hardware.

This study utilizes BMv2 (Behavioral Model Version 2) [15] as the software switch, which supports P4 language configurations. The experimental environment consists of a computer with an Intel i5-13400 CPU (Intel Corporation, Santa Clara, CA, USA), 16 GB of RAM, and Ubuntu 16.04 LTS (Canonical Ltd., London, UK). as the operating system. The network topology is created using Mininet [16], a tool that facilitates the construction of virtual network topologies. Each packet processor in the topology is implemented using BMv2, enabling the completion of this experiment.

**Figure 12:**
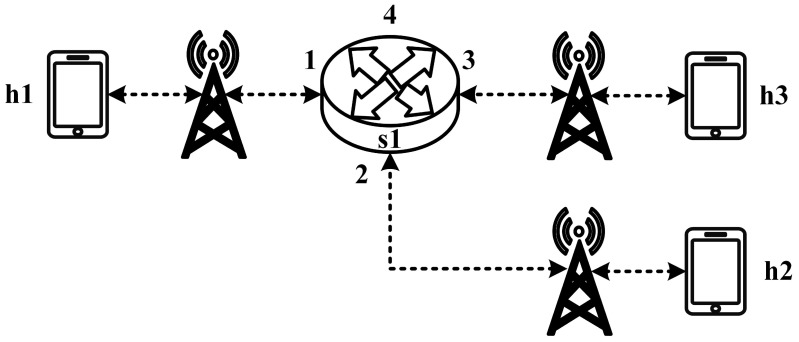
Experimental topology 1.

**Figure 13:**
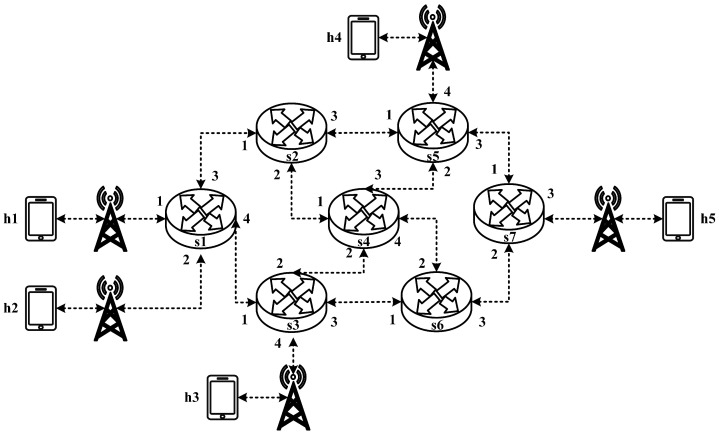
Experimental topology 2.

All experimental topologies in this study are designed under the assumption of a wireless network environment, as shown in Figure 12 and Figure 13. The network consists of seven packet processors and five end devices, with each link having a bandwidth of 10 Mbps. Every packet processor is configured with the same P4 program, which implements the RSVP-supported data plane designed in this study. The system assumes that the RSVP signaling mechanism has already been completed, and the traffic identification and QoS parameters have been directly configured into the data plane parameters.

All experimental traffic is generated using iperf [17], a widely used network performance testing tool. Iperf is chosen due to its support for multiple protocols and its ability to configure transmission duration, bandwidth, and packet size. Additionally, iperf provides essential performance metrics, including throughput and jitter, making it a suitable tool for evaluating network performance. The experiment consists of five parts:

(1) RSVP Meter Comparison—Evaluates the bandwidth allocation before and after enabling rsvp_meter, measuring its effect on RSVP traffic at the receiver;
(2) Non-RSVP Meter Comparison—Assesses the impact of nonrsvp_meter, comparing the bandwidth of non-RSVP traffic before and after applying the meter;
(3) Priority-Based RSVP Traffic Analysis—Tests RSVP traffic with different priority levels and analyzes the impact on packet reception;
(4) Packet Counter Analysis—Monitors the traffic of a specific packet processor, reports the data to the control plane, and helps to determine reasonable bandwidth reservation;
(5) Jitter Analysis with Delay Control—Measures the average jitter variation before and after enabling delay control mechanisms.

These two topologies allow us to verify that our designed system can be used in both a small network with a single switch and a medium-to-large network with seven switches, ensuring the correctness and reliability of our system.

### 5.2. Small-Network Topology Testing

#### 5.2.1. RSVP Meter Comparison

**Figure 14:**
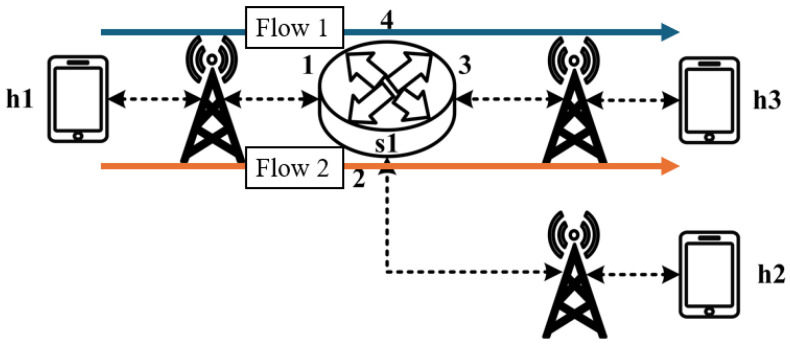
Scenario 1.

The experimental traffic scenarios consists of RSVP-reserved flows, as illustrated in Figure 14. The network topology includes two 8 Mbps UDP traffic flows with the following transmission path:

(1) Flow 1(RSVP traffic): h1 → s1 → h3 (8 Mbps UDP traffic);
(2) Flow 2(RSVP traffic): h2 → s1 → h3 (8 Mbps UDP traffic).

For flow 1, the rsvp_meter is configured with a CIR of 7 Mbps and a PIR of 7.5 Mbps.

For flow 2, the rsvp_meter is configured with a CIR of 2.5 Mbps and a PIR of 3 Mbps.

**Figure 15:**
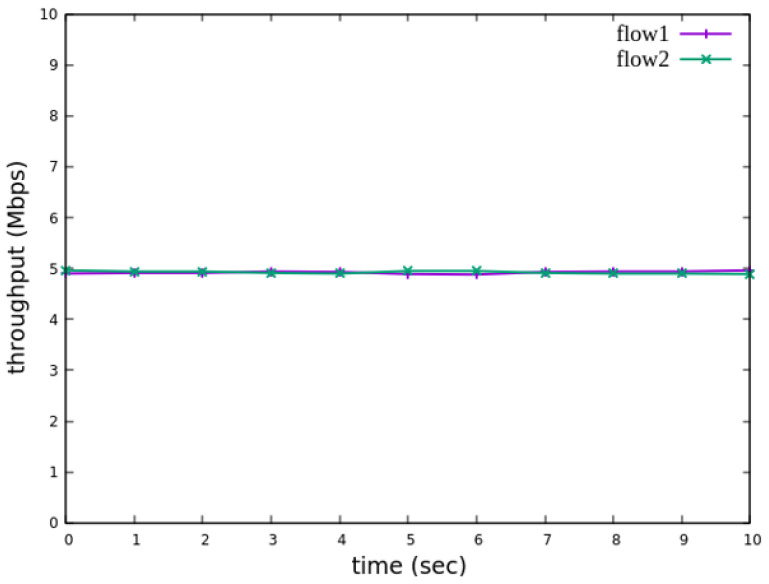
The reception bandwidth at h3 without using rsvp_meter.

**Figure 16:**
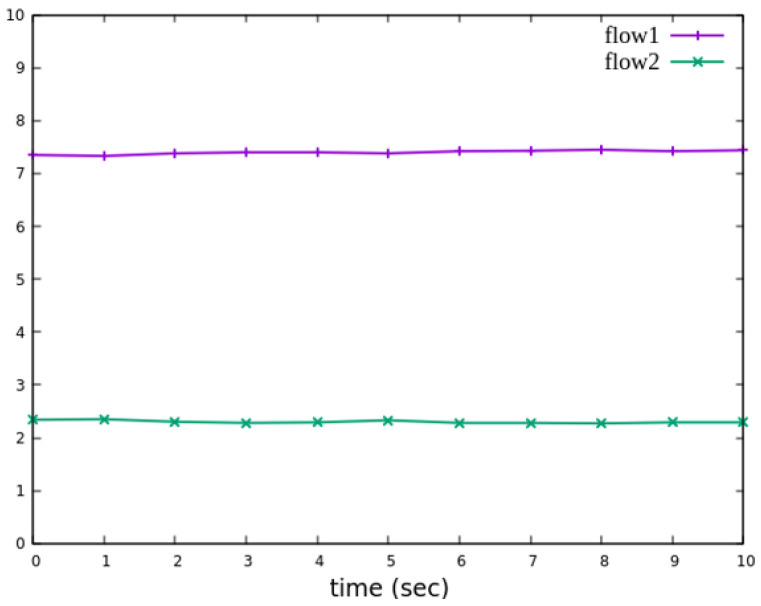
The reception bandwidth at h3 with using rsvp_meter.

Figure 15 and Figure 16 show the reception bandwidth at h3 without and with the rsvp_meter

#### 5.2.2. Non-RSVP Meter Comparison

**Figure 17:**
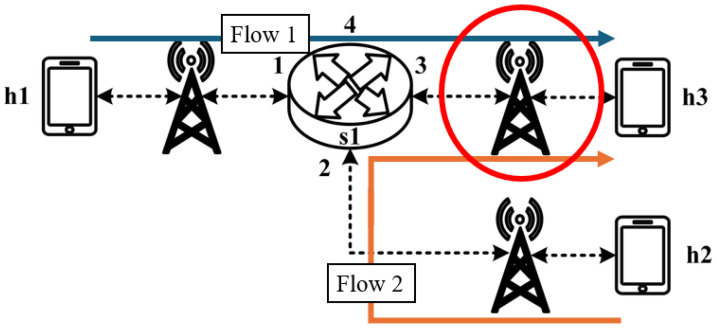
Scenario 2.

As illustrated in Figure 17, the experiment consists of the following traffic flows:

(1) Flow 1 (RSVP Traffic): A 7 Mbps UDP flow following the path h1 → s1 → h3, which has reserved bandwidth through the RSVP signaling mechanism;
(2) Flow 2 (Non-RSVP Traffic): A 7 Mbps UDP flow following the path h2 → s1 → h3, which does not have reserved resources.

When all traffic flows are transmitted simultaneously, the link between s1 and h3 becomes a bottleneck. However, since flow 1 has reserved 7 Mbps of bandwidth through RSVP, the system enforces rate limiting on non-RSVP traffic using the nonrsvp_meter, which is deployed on each packet processor. At switch s1, the system limits the bandwidth of flow 2 (non-RSVP traffic) to 3 Mbps, calculated by subtracting the reserved bandwidth (7 Mbps) from the total link capacity (10 Mbps), leaving 3 Mbps available for non-RSVP traffic.

**Figure 18:**
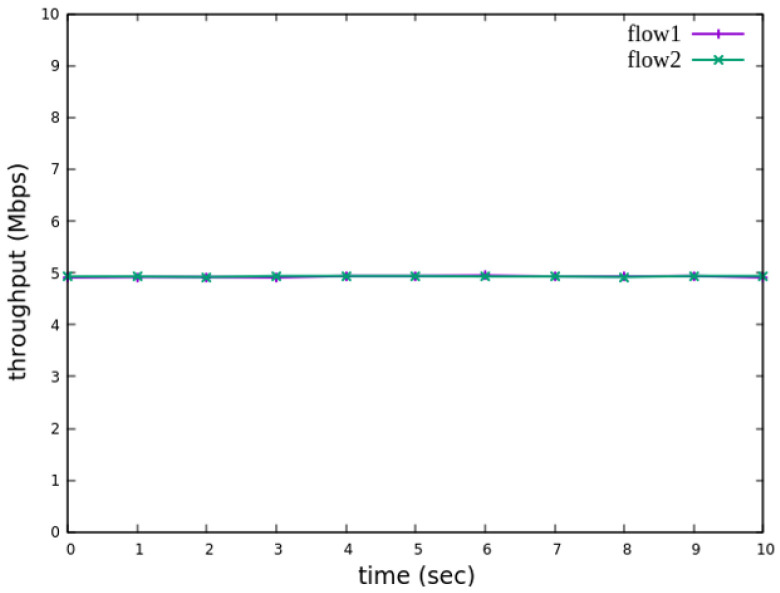
The reception bandwidth at h3 without using nonrsvp_meter.

**Figure 19:**
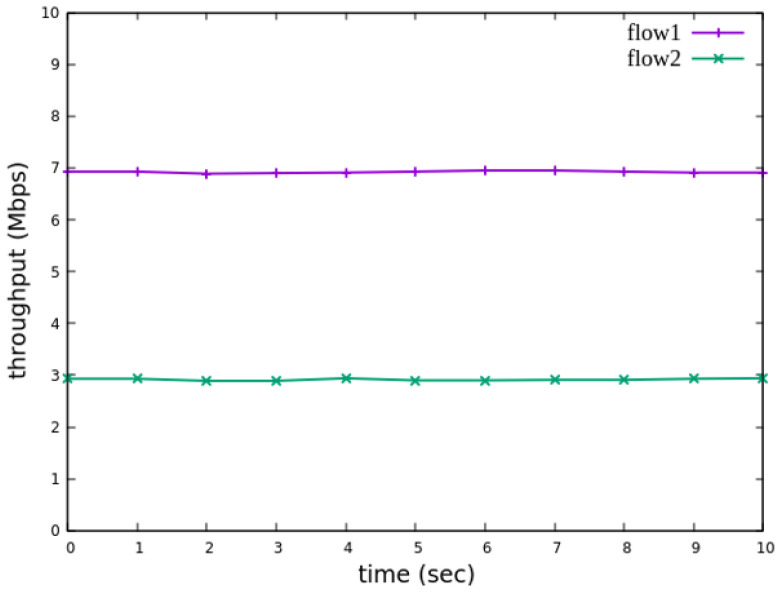
The reception bandwidth at h3 with using nonrsvp_meter.

The experiment monitors flow 1 and flow 2 at receiver h3 to evaluate the effectiveness of bandwidth allocation. The results indicate that, when non-RSVP traffic is not rate-limited, as shown Figure 18, each flow equally shares the 10 Mbps link capacity, with both traffic flows converging toward 5 Mbps. When nonrsvp_meter is applied, it restricts non-RSVP traffic from utilizing bandwidth reserved for RSVP flows. As illustrated in Figure 19, after enforcing rate limits on non-RSVP traffic, RSVP traffic is able to maintain its reserved bandwidth at an average of 7 Mbps, while other traffic is limited to approximately 3 Mbps. These results validate the correct functionality of nonrsvp_meter, ensuring that non-RSVP traffic does not exceed its allocated capacity and that RSVP traffic receives the guaranteed bandwidth as expected.

#### 5.2.3. Priority-Based RSVP Traffic Analysis

**Figure 20:**
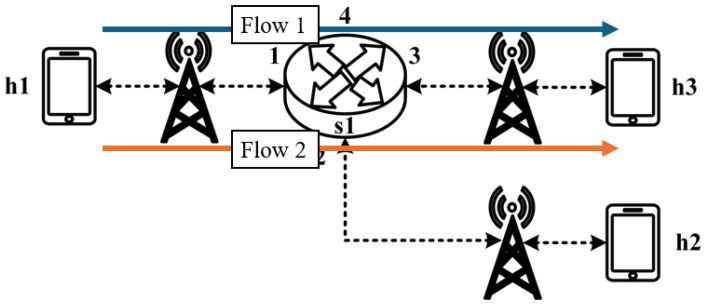
Scenario 3.

As illustrated in Figure 20, this experiment involves the transmission of two RSVP-reserved UDP traffic flows, with the following configurations:

(1) Flow 1 (RSVP Traffic): A 7 Mbps UDP flow traversing the path h1 → s1 → h3;
(2) Flow 2 (RSVP Traffic): Another 7 Mbps UDP flow following the path h1 → s1 → h3.

Although both traffic flows share the same network path, they operate under different application modes, distinguished by their Layer 4 (L4) destination ports. The RSVP metering configuration applies a CIR of 4 Mbps, representing the reserved bandwidth, and a PIR of 6 Mbps, allowing for burst traffic up to 6 Mbps. This experiment compares two scenarios: one where flow 1 is assigned a higher priority than flow 2, and another where both flows are assigned the same priority level. The results assess the impact of priority configurations on RSVP traffic transmission under constrained network conditions.

**Figure 21:**
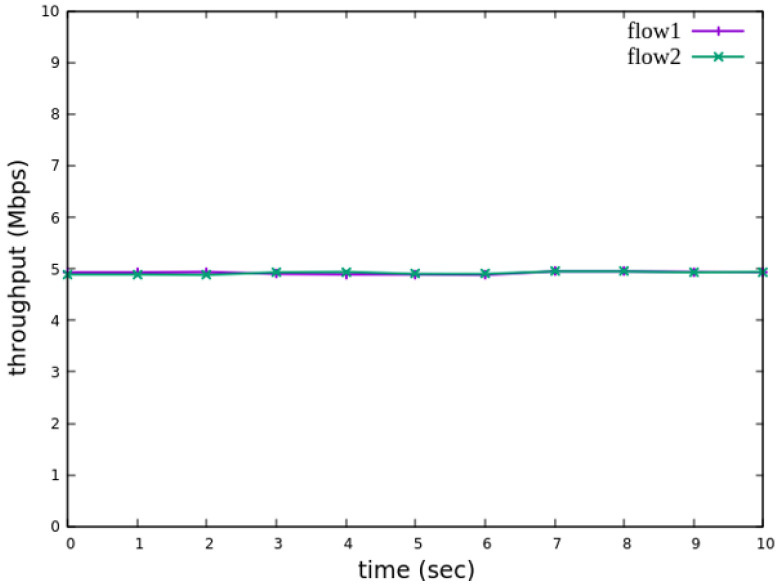
Reception status at h3 without priority configuration.

**Figure 22:**
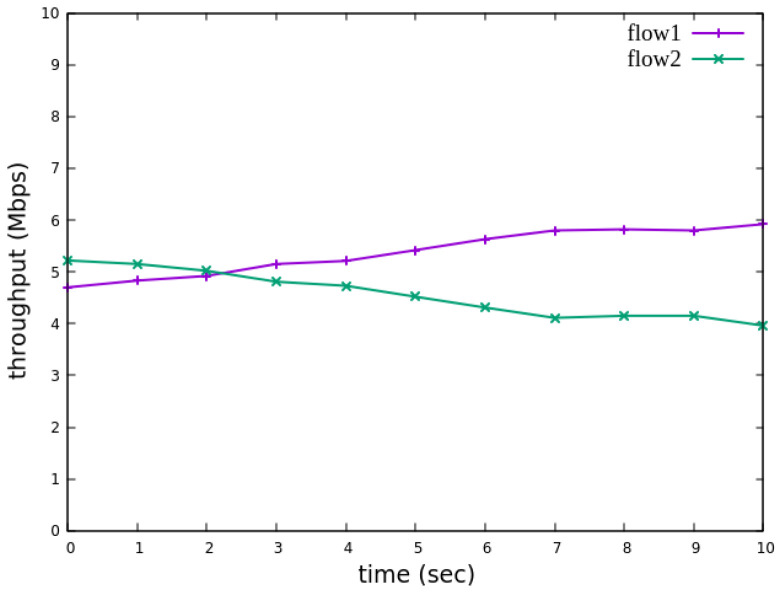
Reception status at h3 with priority configuration.

The experimental results indicate that, when no priority is assigned to RSVP traffic, as shown in Figure 21, the bandwidth monitored at receiver h3 shows that both RSVP flows equally share the link capacity, each receiving approximately 5 Mbps of bandwidth. When flow 1 is assigned a higher priority than flow 2, as illustrated in Figure 22, flow 1 is able to claim the excess bandwidth beyond its guaranteed 4 Mbps, gradually reaching 6 Mbps, while the bandwidth of flow 2 decreases toward 4 Mbps. These results validate the effectiveness of the RSVP priority configuration in this study, confirming that the system correctly prioritizes higher-priority RSVP flows when distributing available bandwidth.

#### 5.2.4. Packet-Counter Analysis

**Figure 23:**
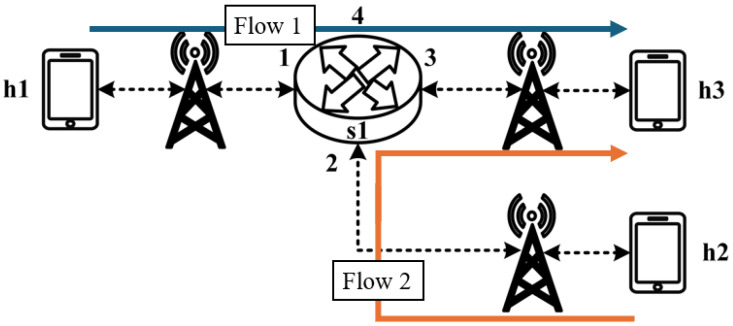
Scenario 4.

Assuming the transmission scenario illustrated in Figure 23, this experiment involves two traffic flows:

(1) Flow 1 (RSVP Traffic): transmitting 3 Mbps UDP traffic along the path h1 → s1 → h3;
(2) Flow 2 (non-RSVP Traffic): transmitting 2 Mbps UDP traffic along the path h1 → s1 → h3.

This experiment is designed to verify whether the system correctly configures and utilizes the counter function. The counter is used to monitor the usage of the general network resources, providing insight to the control plane. By referencing this information, the control plane can make more informed and reasonable decisions when allocating network resources for RSVP reservations.

**Figure 24:**
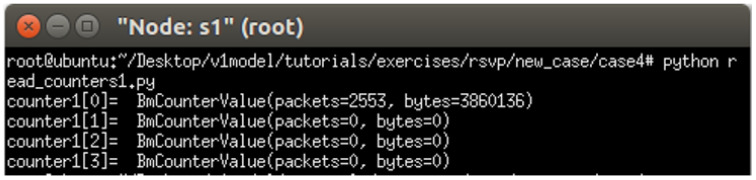
Results of counter1 on s1.

The experiment monitors switch s1, where RSVP and non-RSVP traffic are recorded using two separate counters. Counter 1 is responsible for recording RSVP traffic, while Counter 2 tracks non-RSVP traffic. As shown in Figure 24, by assuming that the RSVP session index is 0, the recorded values in Counter 1 (index 0) correspond to flow 1, displaying the total number of packets and bytes transmitted over 10 s at 3 Mbps.

**Figure 25:**
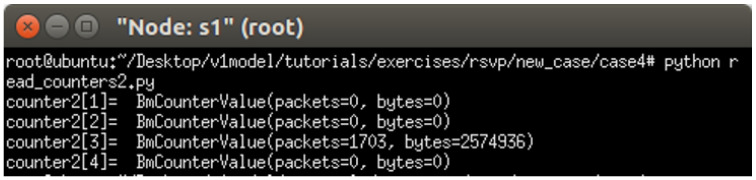
Results of counter2 on s1.

For flow 2, which is transmitted via port 3 of s1, the corresponding data are recorded in counter 2 (index 3). As illustrated in Figure 25, the counter correctly logs the total number of packets and bytes for 10 s of 2 Mbps UDP traffic. Since the recorded counter values precisely match the transmitted data, the experiment verifies the accuracy and correctness of the counter functionality designed in this study.

#### 5.2.5. Jitter Analysis with Delay Control

**Figure 26:**
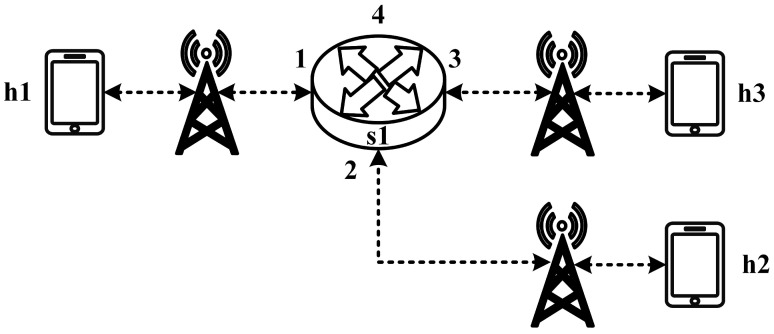
Scenario 5.

Assuming the transmission scenario illustrated in Figure 26, the experiment involves transmitting a single RSVP flow with a transmission path of h1 → s1 → h3 carrying 4 Mbps UDP traffic. The purpose of this experiment is to verify whether the system can effectively enforce delay control mechanisms within packet processors. To evaluate the system’s ability to limit in-device processing delay, the maximum delay threshold is set to 2.5 ms. This ensures that packets exceeding this threshold are dropped. The experiment measures whether the implemented delay control function operates correctly and enforces the specified threshold within the system.

**Figure 27:**
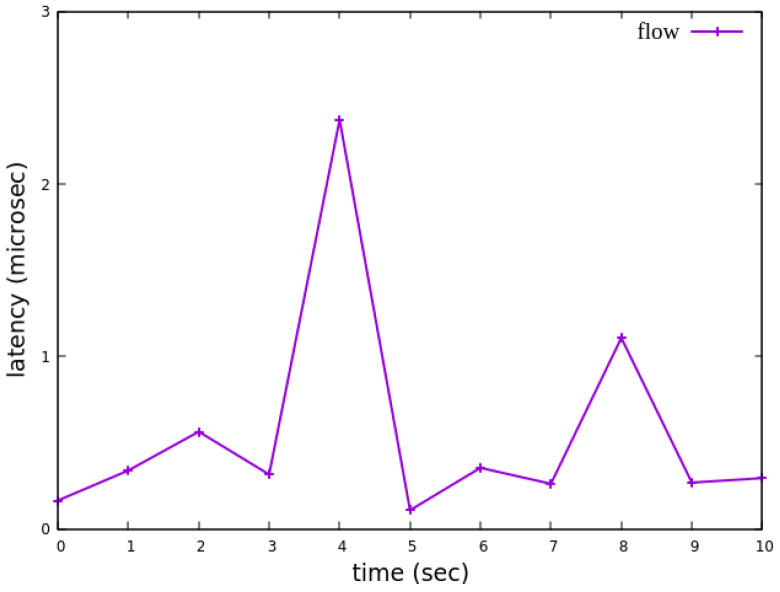
The reception jitter at receiver h3 without latency management.

**Figure 28:**
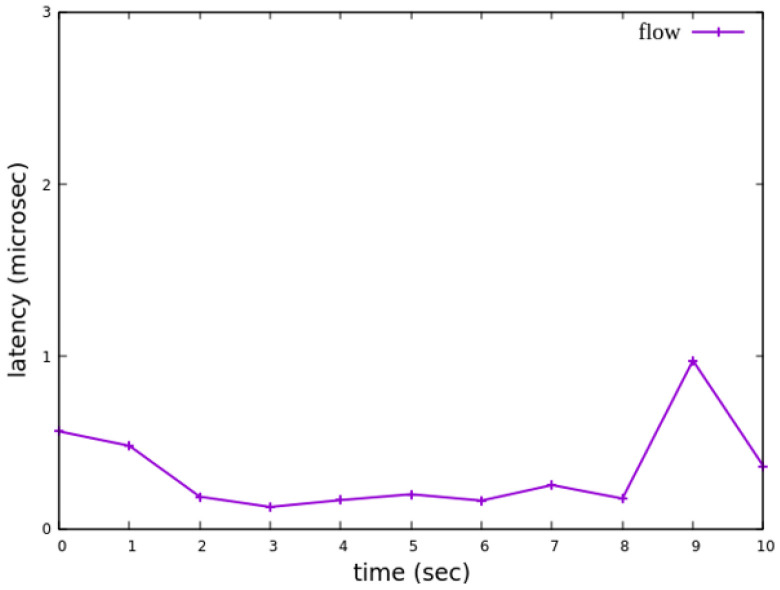
The reception jitter at receiver h3 with latency management.

As shown in Figure 27, when delay control is not applied, the jitter exhibits extreme values at the third and eighth seconds. These fluctuations indicate instability in packet transmission latency. After applying delay control to the traffic, as illustrated in Figure 28, the overall jitter values become significantly more stable compared to Figure 27, with extreme values completely eliminated. This result demonstrates that the delay control mechanism effectively smooths out jitter, ensuring more consistent packet transmission latency.

### 5.3. Medium-to-Large Network Topology Testing

#### 5.3.1. RSVP Meter Comparison

**Figure 29:**
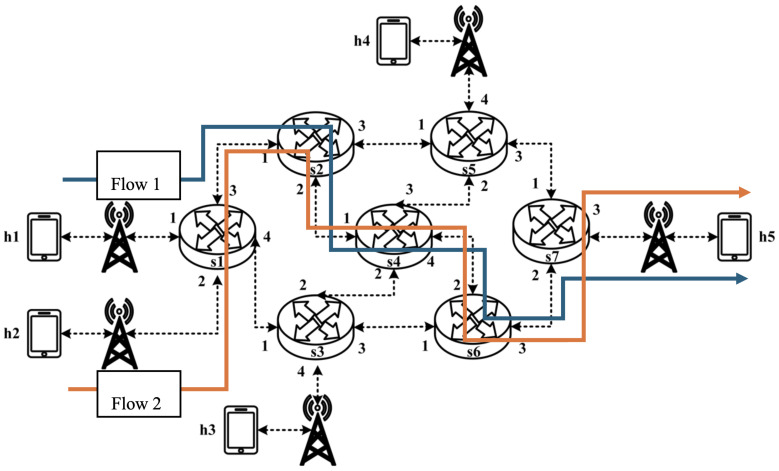
Scenario 6.

The experimental traffic consists of RSVP-reserved flows, as illustrated in Figure 29. The network topology includes two 8 Mbps UDP traffic flows with the following transmission path:

(1) Flow 1: h1 → s1 → s2 → s4 → s6 → s7 → h5 (8 Mbps UDP traffic);
(2) Flow 2: h2 → s1 → s2 → s4 → s6 → s7 → h5 (8 Mbps UDP traffic).

For flow 1, the rsvp_meter is configured with a CIR of 6 Mbps and a PIR of 6.5 Mbps.

For flow 2, the rsvp_meter is configured with a CIR of 3.5 Mbps and a PIR of 4 Mbps.

**Figure 30:**
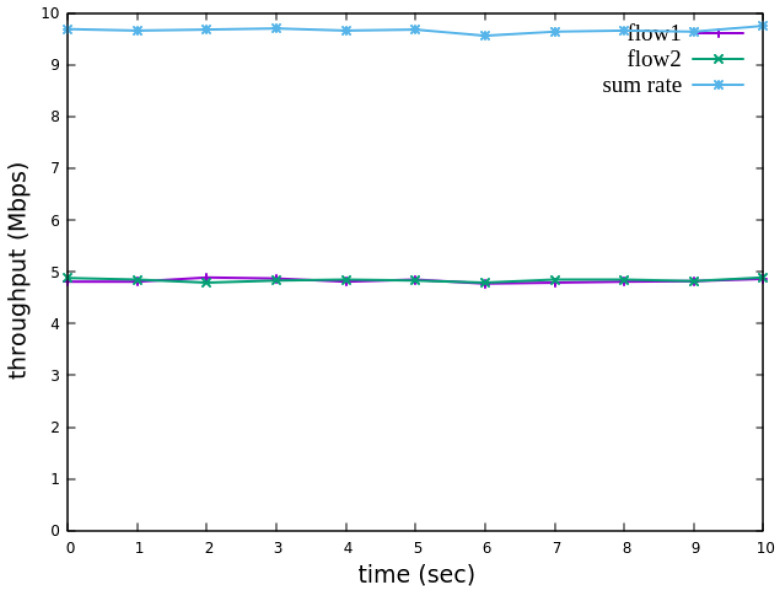
The reception bandwidth at h5 without using rsvp_meter.

**Figure 31:**
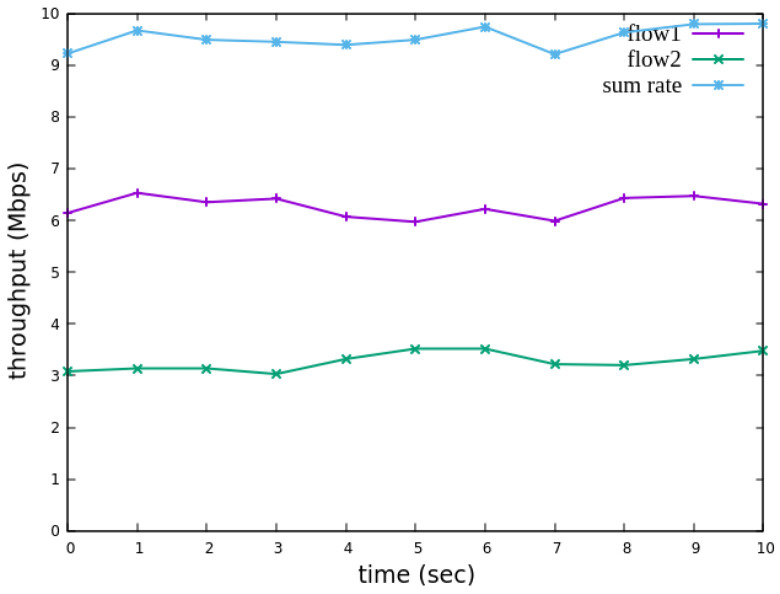
The reception bandwidth at h5 with using rsvp_meter.

As illustrated in Figure 30, when PIR and CIR are not configured for rsvp_meter, the monitored traffic at receiver h5 shows that the received bandwidth is evenly distributed, with both flows converging toward 5 Mbps. When flow 1 is configured with a CIR of 6 Mbps and a PIR of 6.5 Mbps and when flow 2 is configured with a CIR of 3.5 Mbps and a PIR of 4 Mbps, the results in Figure 31 indicate that the received bandwidth at h5 falls within the configured limits. This confirms that the rsvp_meter is correctly implemented, effectively regulating traffic according to the assigned rate parameters.

#### 5.3.2. Non-RSVP Meter Comparison

**Figure 32:**
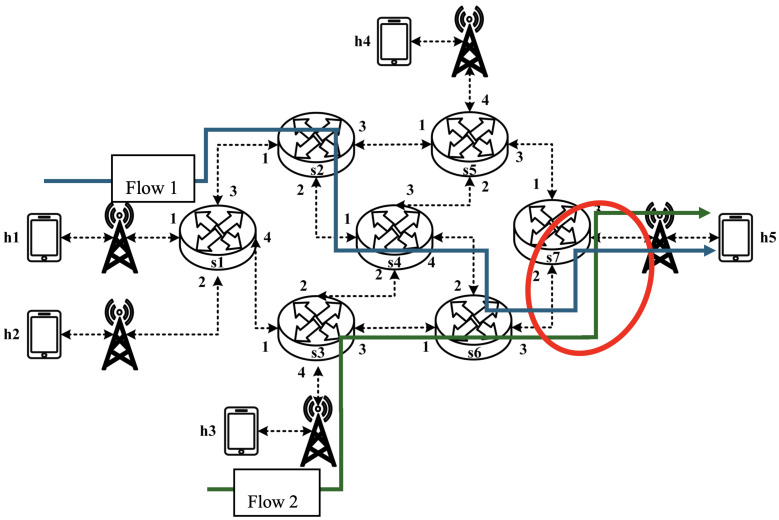
Scenario 7.

As illustrated in Figure 32, the experiment consists of the following traffic flows:

(1) Flow 1 (RSVP Traffic): A 6 Mbps UDP flow following the path h1 → s1 → s2 → s4 → s6 → s7 → h5, which has reserved bandwidth through the RSVP signaling mechanism;
(2) Flow 2 (Non-RSVP Traffic): A 6 Mbps UDP flow following the path h3 → s3 → s6 → s7 → h5, which does not have reserved resources.

When all traffic flows are transmitted simultaneously, the link between s6 and h5 becomes a bottleneck. However, since flow 1 has reserved 6 Mbps of bandwidth through RSVP, the system enforces rate limiting on non-RSVP traffic using the nonrsvp_meter, which is deployed on each packet processor. At switch s6, the system limits the bandwidth of flow 2 (non-RSVP traffic) to 4 Mbps, calculated by subtracting the reserved bandwidth (6 Mbps) from the total link capacity (10 Mbps), leaving 4 Mbps available for non-RSVP traffic.

**Figure 33:**
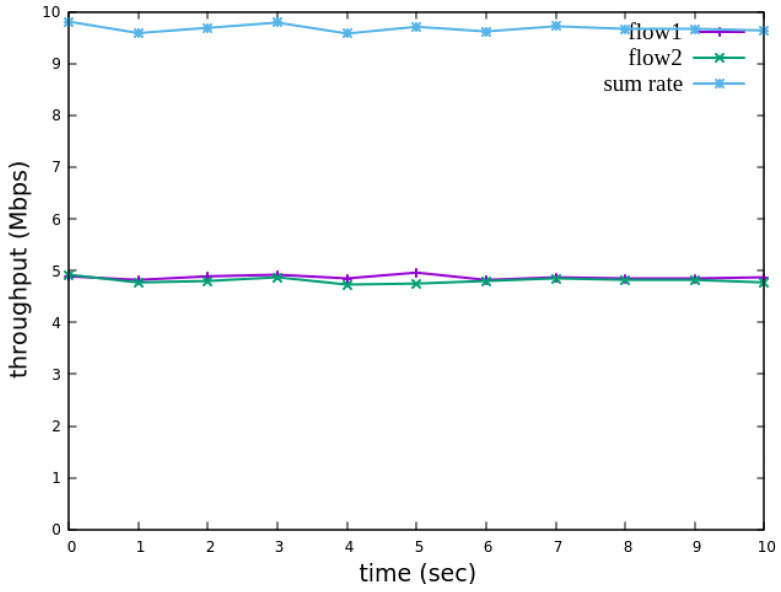
The reception bandwidth at h5 without using nonrsvp_meter.

**Figure 34:**
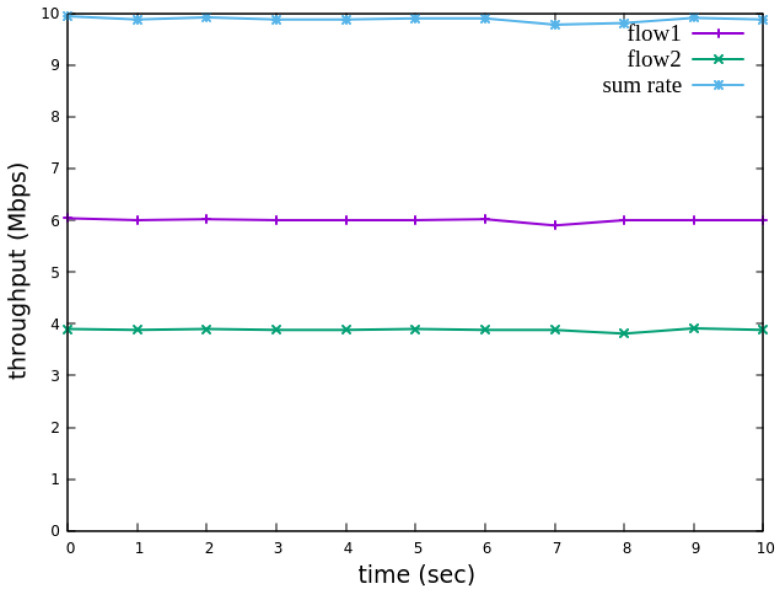
The reception bandwidth at h5 with using nonrsvp_meter.

The experiment monitors flow 1 and flow 2 at receiver h5 to evaluate the effectiveness of bandwidth allocation. The results indicate that, when non-RSVP traffic is not rate-limited, as shown Figure 33, each flow equally shares the 10 Mbps link capacity, with both traffic flows converging toward 5 Mbps. When nonrsvp_meter is applied, it restricts non-RSVP traffic from utilizing bandwidth reserved for RSVP flows. As illustrated in Figure 34, after enforcing rate limits on non-RSVP traffic, RSVP traffic is able to maintain its reserved bandwidth at an average of 6 Mbps, while other traffic is limited to approximately 4 Mbps. These results validate the correct functionality of nonrsvp_meter, ensuring that non-RSVP traffic does not exceed its allocated capacity and that RSVP traffic receives the guaranteed bandwidth as expected.

#### 5.3.3. Priority-Based RSVP Traffic Analysis

**Figure 35:**
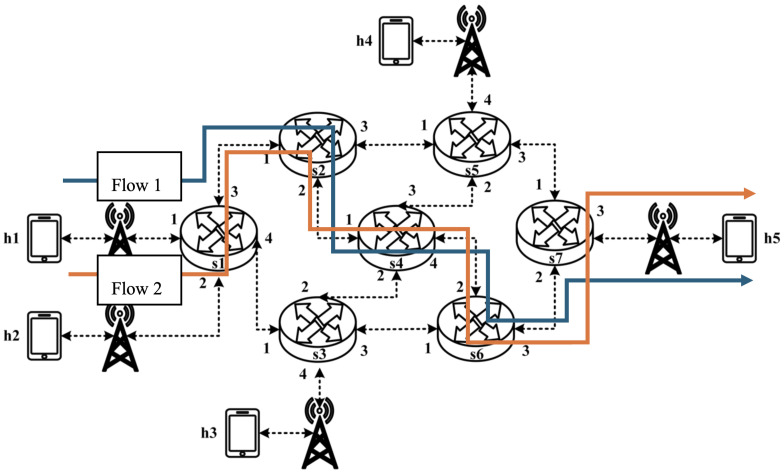
Scenario 8.

As illustrated in Figure 35, the experiment involves the transmission of two RSVP-reserved UDP traffic flows, with the following configurations:

(1) Flow 1 (RSVP Traffic): An 8 Mbps UDP flow traversing the path h1 → s1 → s2 → s4 → s6 → s7 → h5;
(2) Flow 2 (RSVP Traffic): Another 8 Mbps UDP flow following the path h1 → s1 → s2 → s4 → s6 → s7 → h5.

Although both traffic flows share the same network path, they operate under different application modes, distinguished by their Layer 4 (L4) destination ports. The RSVP metering configuration applies a CIR of 4 Mbps, representing the reserved bandwidth, and a PIR of 6 Mbps, allowing for burst traffic up to 6 Mbps. This experiment compares two scenarios: one where flow 1 is assigned a higher priority than flow 2, and another where both flows are assigned the same priority level. The results assess the impact of priority configurations on RSVP traffic transmission under constrained network conditions.

**Figure 36:**
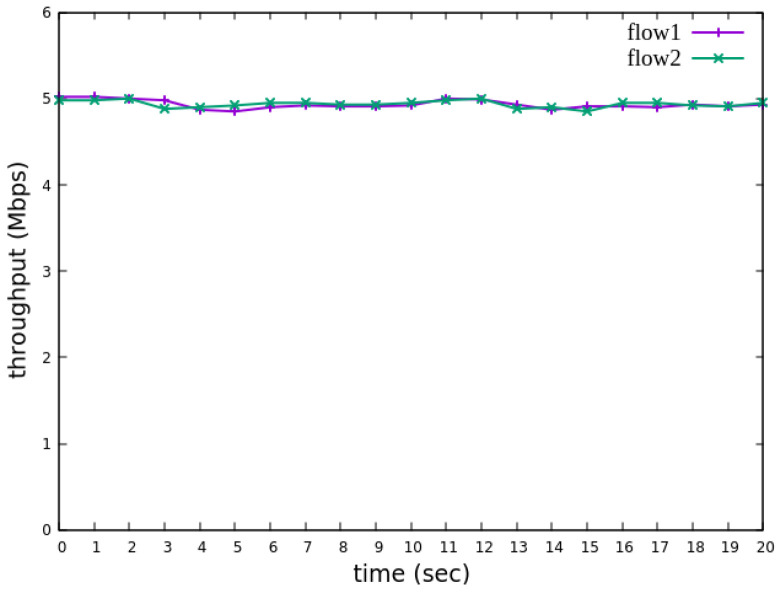
Reception status at h5 without priority configuration.

**Figure 37:**
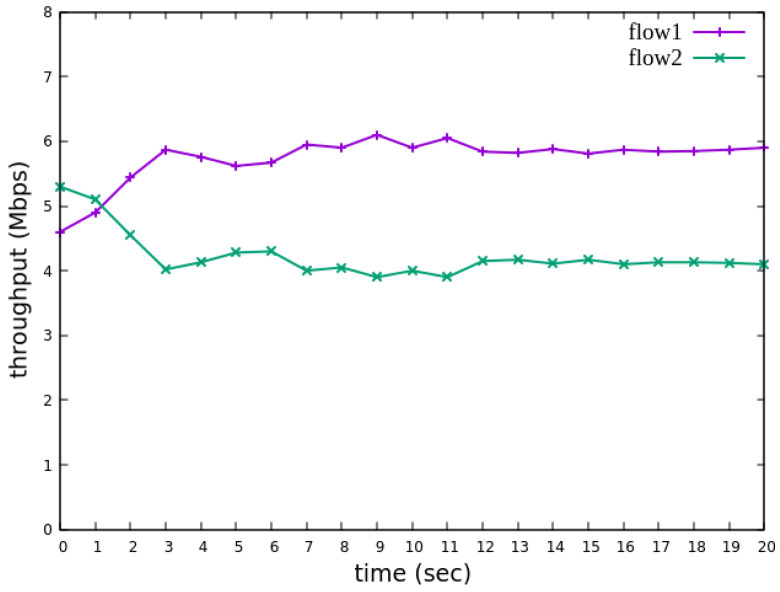
Reception status at h5 with priority configuration.

The experimental results indicate that, when no priority is assigned to RSVP traffic, as shown in Figure 36, the bandwidth monitored at receiver h5 shows that both RSVP flows equally share the link capacity, each receiving approximately 5 Mbps of bandwidth. When flow 1 is assigned a higher priority than flow 2, as illustrated in Figure 37, flow 1 is able to claim the excess bandwidth beyond its guaranteed 4 Mbps, gradually reaching 6 Mbps, while the bandwidth of flow 2 decreases toward 4 Mbps. These results validate the effectiveness of the RSVP priority configuration in this study, confirming that the system correctly prioritizes higher-priority RSVP flows when distributing available bandwidth.

#### 5.3.4. Packet-Counter Analysis

**Figure 38:**
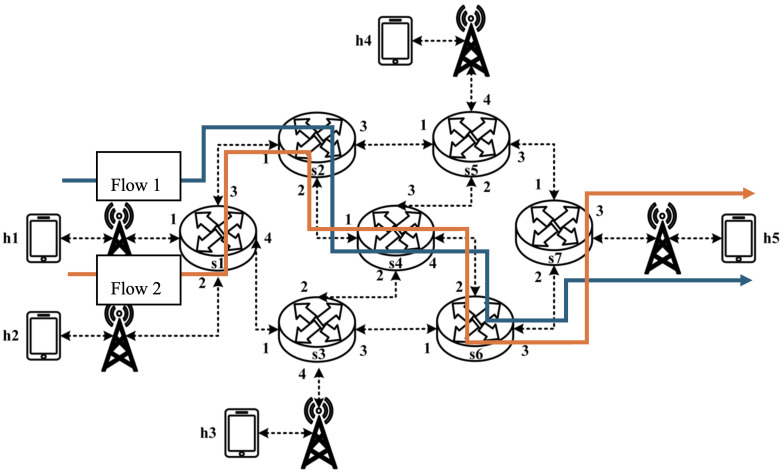
Scenario 9.

When assuming the transmission scenario illustrated in Figure 38, the experiment involves two traffic flows:

(1) Flow 1 (RSVP Traffic): transmitting 4 Mbps UDP traffic along the path h1 → s1 → s2 → s4 → s6 → s7 → h5;
(2) Flow 2 (non-RSVP Traffic): transmitting 3 Mbps UDP traffic along the path h1 → s1 → s2 → s4 → s6 → s7 → h5.

This experiment is designed to verify whether the system correctly configures and utilizes the counter function. The counter is used to monitor the usage of the general network resources, providing insight to the control plane. By referencing this information, the control plane can make more informed and reasonable decisions when allocating network resources for RSVP reservations.

**Figure 39:**
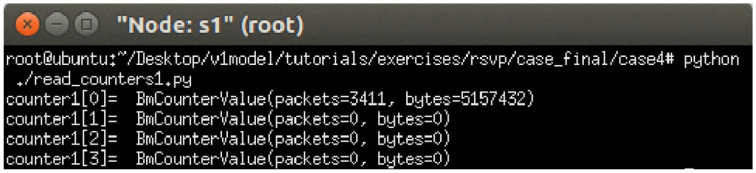
Results of counter1 on s1.

The experiment monitors switch s1, where RSVP and non-RSVP traffic are recorded using two separate counters. Counter 1 is responsible for recording RSVP traffic, while Counter 2 tracks non-RSVP traffic. As shown in Figure 39, by assuming that the RSVP session index is 0, the recorded values in Counter 1 (index 0) correspond to flow 1, displaying the total number of packets and bytes transmitted over 10 s at 4 Mbps.

**Figure 40:**
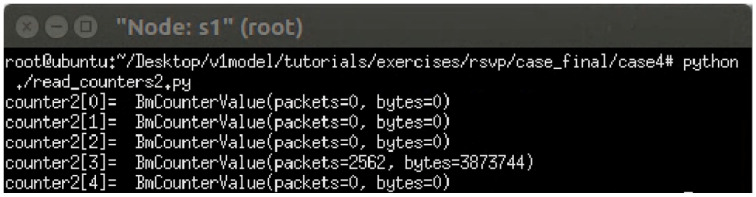
Results of counter2 on s1.

For flow 2, which is transmitted via port 3 of s1, the corresponding data are recorded in Counter 2 (index 3). As illustrated in Figure 40, the counter correctly logs the total number of packets and bytes for 10 s of 3 Mbps UDP traffic. Since the recorded counter values precisely match the transmitted data, the experiment verifies the accuracy and correctness of the counter functionality designed in this study.

#### 5.3.5. Jitter Analysis with Delay Control

**Figure 41:**
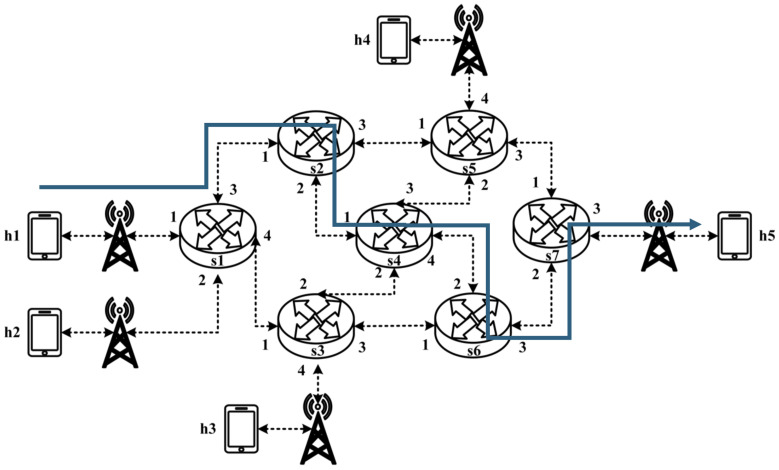
Scenario 10.

When assuming the transmission scenario illustrated in Figure 41, the experiment involves transmitting a single RSVP flow with a transmission path of h1 → s1 → s2 → s4 → s6 → s7 → h5 carrying 4 Mbps UDP traffic. The purpose of this experiment is to verify whether the system can effectively enforce delay control mechanisms within packet processors. To evaluate the system’s ability to limit in-device processing delay, the maximum delay threshold is set to 3.85 ms. This ensures that packets exceeding this threshold are dropped. The experiment measures whether the implemented delay control function operates correctly and enforces the specified threshold within the system.

**Figure 42:**
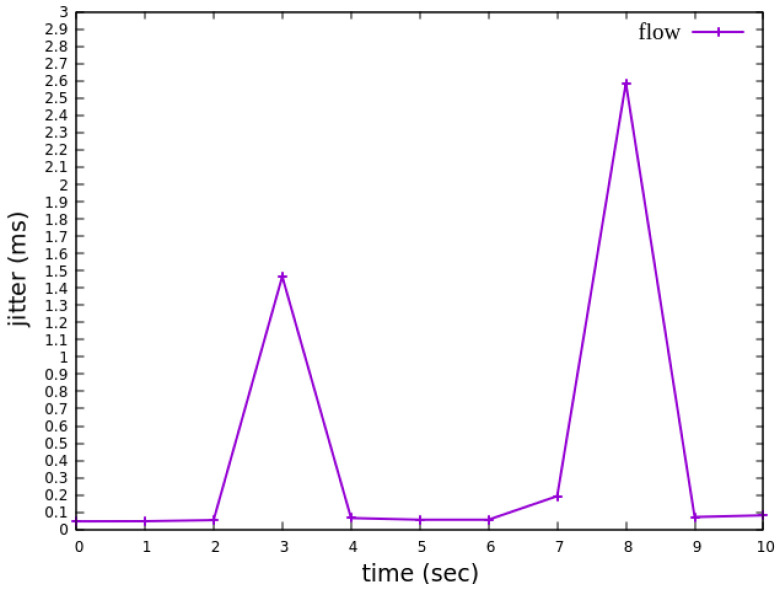
The reception jitter at receiver h5 without latency management.

**Figure 43:**
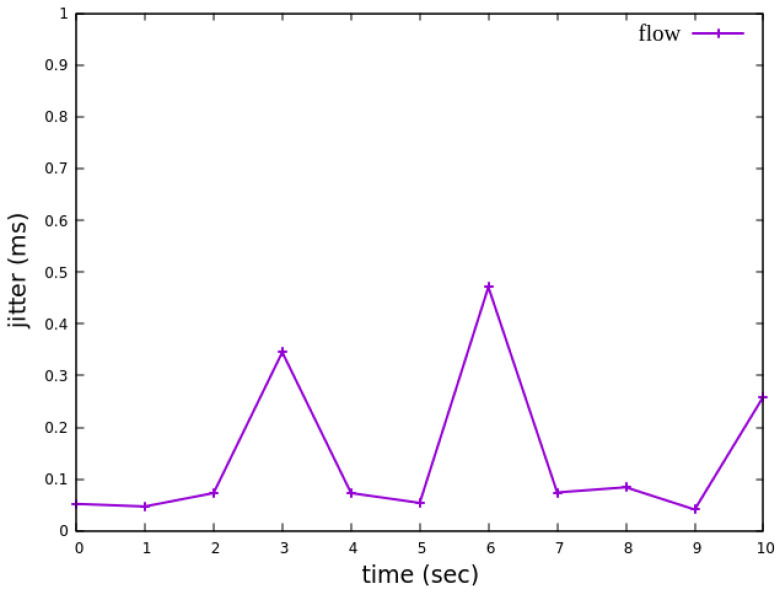
The reception jitter at receiver h5 with latency management.

As shown in Figure 42, when delay control is not applied, the jitter exhibits extreme values at the third and eighth seconds. These fluctuations indicate instability in packet transmission latency. After applying delay control to the traffic, as illustrated in Figure 43, the overall jitter values become significantly more stable compared to Figure 42, with extreme values completely eliminated. This result demonstrates that the delay control mechanism effectively smooths out jitter, ensuring more consistent packet transmission latency.

## 6. Conclusions

This study explores the data plane configuration requirements to support the RSVP protocol, including identifying RSVP traffic and ensuring its QoS requirements. A P4-based data plane was designed using V1Model, implementing RSVP-specific functionalities. The design includes logic for each block of V1Model: the Parser block processes RSVP packet parsing; the Ingress match-action block retrieves RSVP parameters for bandwidth and priority requirements and implements metering, counters, priority configurations, and packet field modifications. These components, respectively, ensure bandwidth reservation for RSVP traffic, prevent excessive resource usage by non-RSVP traffic, assign different priority levels to RSVP flows, and monitor network transmission conditions. In the Egress match-action block, RSVP delay requirements are addressed by leveraging P4 standard metadata timestamps; the difference between the Egress and Ingress timestamps is used to discard packets that exceed the processing time threshold.

A virtual wireless network topology was established using Mininet, with configured BMv2 software packet processors. Iperf was used to generate traffic to test RSVP-related QoS requirements implemented in the data plane, evaluating the accuracy of the priority configuration, RSVP-specific metering, non-RSVP metering, latency management, and counters. Ten experiments were designed to evaluate these QoS mechanisms, ultimately validating the correctness of the RSVP packet processing logic implemented using P4. Furthermore, this study assumes that the network is deployed in both wired and wireless environments, ensuring that RSVP-based QoS guarantees can also be applied in wireless networks, where managing bandwidth and delay is crucial due to varying network conditions.

This study validates the powerful potential of the P4 framework in network design, demonstrating its flexibility in QoS assurance and resource management. By implementing the RSVP reservation mechanism in the P4 data plane, this study proves that P4 switches can efficiently perform bandwidth allocation, delay control, and priority configuration, laying the foundation for more fine-grained QoS strategies in future programmable network environments.

Furthermore, to verify the applicability and reliability of the proposed approach across networks of different scales, we designed a small network with a single switch and a medium-to-large network consisting of seven switches, evaluating their QoS performance. Experimental results show that, regardless of network complexity, the proposed RSVP-P4 mechanism effectively maintains the expected QoS guarantees, ensuring service quality across different nodes and demonstrating excellent scalability and adaptability.

At the same time, this study highlights the versatility of P4 as a network programming framework, enabling its application across various network architectures. This underscores P4’s broad potential for future network innovation and optimization.

The previous study [7] pointed out that the TCP ECN mechanism and the P4 meter mechanism may conflict, leading to unstable traffic behavior, particularly when the TCP congestion control mechanism excessively reduces the transmission rate due to packet drops or markings by the meter, thereby affecting the performance of the QoS reservation mechanism. The main objective of this study is to validate the QoS control capability of the P4 data plane under the RSVP resource reservation mechanism. Therefore, a more stable UDP traffic flow was used in the experimental design to ensure that the test results accurately reflect the impact of P4 switches on bandwidth allocation, delay control, and priority configuration.

However, TCP is widely used in practical network environments, and ensuring the adaptability of the RSVP-P4 mechanism to TCP traffic remains a critical issue. Therefore, future research will further explore the behavior of TCP traffic under this mechanism and evaluate the competition between TCP and UDP in a QoS reservation environment. Additionally, we will explore more suitable congestion management strategies for TCP, such as combining ECN and RED (Random Early Detection) mechanisms, to replace the traditional P4 meter-based packet drop approach. This will ensure that TCP traffic can stably and fairly utilize the reserved bandwidth, improving the applicability and stability of the P4-based QoS mechanism in heterogeneous traffic environments.

For future work, the P4-utils library could be used to develop Python-based control scripts, implementing the complete RSVP signaling mechanism. By integrating this control plane with the P4-based data plane designed in this study, RSVP can be fully deployed within an SDN-based network architecture, supporting diverse and complex application scenarios, including wireless networks, where dynamic resource management and adaptive QoS provisioning are essential. This study can further explore the integration of P4 Runtime to achieve a more flexible dynamic control mechanism. Currently, flow table entries and meter parameters are manually configured using simple_switch_CLI to test the QoS management mechanisms of the P4 data plane. In the future, P4 Runtime could be introduced to enable the control plane to automatically update table entries and bandwidth management parameters based on real-time network conditions, reducing manual intervention and improving system adaptability. Additionally, integration with existing SDN control frameworks (such as ONOS or Stratum) could be explored to achieve cross-device QoS management and ensure a more comprehensive end-to-end resource reservation mechanism. Through these optimizations and extensions, the proposed approach could be more widely applied to different network environments, enhancing the flexibility and applicability of P4 switches in QoS assurance.

## Data Availability

Data are contained within the article.

## Abbreviations

The following abbreviations are used in this manuscript:

SDN	Software-Defined Networking
P4	Programming Protocol-Independent Packet Processors
RSVP	Resource Reservation Protocol
QoS	Quality of Service
